# Vertically Aligned Nanopillar Electrodes: Engineered Interfaces for Electrophysiology and Cell‐Electrode Coupling

**DOI:** 10.1002/smtd.70728

**Published:** 2026-05-24

**Authors:** Mohammad Alzahrani, Ismat Kabbara, Shuhua Peng, Hoang‐Phuong Phan, Tushar Kumeria

**Affiliations:** ^1^ School of Mechanical and Manufacturing Engineering the University of New South Wales Sydney New South Wales Australia; ^2^ School of Materials Science and Engineering University of New South Wales Sydney New South Wales Australia; ^3^ Australian Centre for Nanomedicine University of New South Wales Sydney New South Wales Australia

**Keywords:** biophysical measurements, electrophysiological sensors, nanopillar electrodes, nanowire electrodes, porous templates

## Abstract

Vertically aligned nanopillar structures have been extensively studied and exploited in numerous applications across various fields due to their unique features, including high surface area and aspect ratio, small size, superior electrical, mechanical, and optical characteristics. Of particular interest are their interesting interfacial properties, which make them ideal for use as electrodes for biophysiological measurements. This comprehensive review explores the growing applications and fabrication techniques of vertically aligned nanopillar electrodes, emphasizing their functions in electrophysiological sensing particularly. The review begins with an overview of the biophysical measurements offered by these electrodes, including electrocardiogram (ECG), electroencephalography (EEG), electromyography (EMG), electrooculography (EOG), and temperature monitoring. Invasive and non‐invasive vertically aligned nanopillar electrodes are classified and evaluated based on characteristics such as conductivity, adhesion, breathability, biocompatibility, biodegradability, and flexibility. Various material systems are considered, including metal‐based, carbon‐based, and polymer‐based electrodes, each contributing distinct advantages to overall performance. Fabrication methods, particularly lithography and template‐assisted synthesis using porous anodic alumina (PAA) and porous silicon (pSi), are highlighted for their precision in controlling geometry and surface properties. The review concludes with an assessment of practical applications, emphasizing the enhanced capabilities of vertically aligned nanopillar electrodes in electrophysiological measurements.

## Graphical abstract

The graphic provides an overview of the emerging field of high‐surface area vertically aligned nanopillar electrodes for electrophysiological measurements. It depicts various types of electrodes; materials, and the methods used for fabrication of vertically aligned nanopillar electrodes. Lastly, various biomedical electrophysiological applications of vertically aligned nanopillar electrodes.

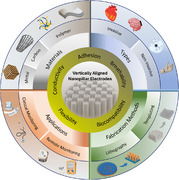

## 1 Introduction

Activities and functions of organs and tissues throughout the human body are controlled by alterations in electrical potentials referred to as electrophysiological (EP) signals [1]. Electrophysiological signals play a crucial role in regulating the functions and operations of tissues and organs within the human nervous system [2]. The electrically active cells throughout the body persistently transport electrical signals, resulting in a persistently fluctuating electric field covering the whole interior space of bodies. As a result, it is possible to detect electrophysiological signals produced from different tissues and organs within the human body [3, 4]. Detecting these signals has the potential to reflect the physiological condition of the human body. It provides crucial information in designing time‐bound, and accurate treatment strategies personalized for the patient [3, 4, 5].

The use of biophysical measurements for diagnostics and human health monitoring purposes has transformed healthcare. The major electrophysiological techniques are electrocardiogram (ECG), electroencephalogram (EEG), electromyogram (EMG), electrooculography (EOG) [6], and electrogastrography (EGG). As their names suggest, these techniques measure the driving electrical signals from various organs. For example, an ECG is a considerably efficient and convenient diagnostic technique performed to examine a wide spectrum of cardiac irregularities [7]. While, the EEG is a commonly employed technique for noninvasively monitoring brain activity [8] and can be utilized in multiple health fields, including fatigue detection, surveillance of sleep, and mental health disorder [9]. EMG can serve as a reliable measurement of muscle function in specific muscle groups following initial injury to the neuromuscular system and during the entire process of rehabilitation [10].

From early disease detection to personalized interventions, the integration of electrophysiological measurements holds strong promise for transforming healthcare practice and enabling dynamic health assessment [11]. Although early electrophysiological measurement systems often relied on invasive procedures to position electrodes near the target organ, the widespread use of modern electrophysiological techniques is largely driven by their ability to provide reliable and non‐invasive assessment of organ health [12]. As such, these techniques have become a cornerstone of healthcare and are widely used for disease diagnosis and patient monitoring in both hospital and home settings.

Accurate real‐time acquisition of electrophysiological signals remains technically challenging because these signals often exhibit low amplitudes, are highly susceptible to noise and external interference, and are spatially distributed [13]. In non‐invasive recordings, these signals undergo additional attenuation and distortion as they propagate through intervening biological layers including skin, bone, and bodily fluids, which introduce impedance mismatches and frequency‐dependent filtering effects [13]. Consequently, reliable extraction of physiologically relevant information requires electrodes that exhibit high sensitivity, low noise characteristics, and stable tissue‐electrode interfaces. Addressing these challenges has required sustained advancements over several decades in electrode materials, surface and interface engineering, and signal conditioning methodologies [14]. Over the years, we have gone from bulky, wired systems to more flexible, wearable, skin‐conformal, ultrathin, self‐healing, non‐invasive, and disposable electrodes. Researchers have created highly sensitive and bendable biophysical electrodes utilizing advanced materials including nanoparticles, nanofibers, sponges, nanowires and pillars, conducting polymers and, hydrogels. A key goal of these efforts is to improve electrical performance without compromising other features like adhesion and wearability [14].

Vertically aligned nanopillar‐based electrodes have demonstrated significant potential for improving signal acquisition and enhancing interfacial adhesion with biological tissues [15]. These advances have accelerated their adoption in emerging applications such as wearable health monitoring and reliable at‐home diagnostic systems. In this review, nanopillars are defined as vertically oriented nanoscale columnar structures anchored to a substrate, typically possessing diameters in the tens to hundreds of nanometers and heights ranging from several hundred nanometers to a few micrometers, resulting in moderate to high aspect ratios [16]. Such architectures provide stable electrical interfaces and increased surface interaction with biological systems. In this review, the term nanopillar is used broadly to describe vertically oriented nanostructures that interface with biological systems, as terminology in the literature is not always applied consistently. In some cases, high–aspect ratio nanostructures that exhibit a slight tilt or angular deviation from the surface normal are still described as vertically aligned nanopillars. It is also worth noting that in certain studies, particularly those involving ultra–high aspect ratio structures, such features are referred to as nanowires. However, for the sake of consistency throughout this review, these structures are also referred to as nanopillars.

**FIGURE 1:**
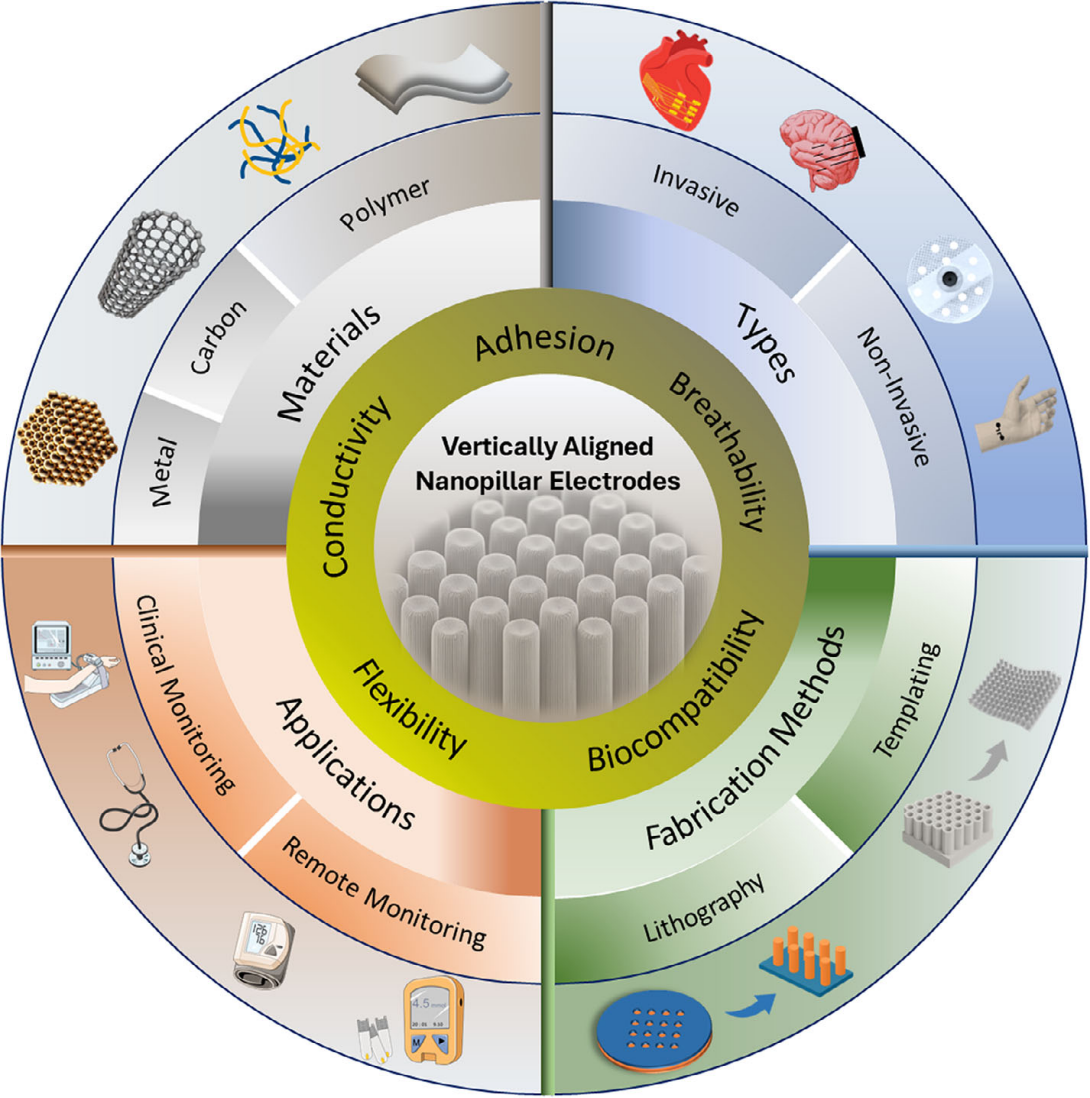
The materials, types, fabrication methods, and applications of nanopillar electrodes for biophysical measurements. Reproduced with permission under a Creative Commons CC BY 4.0 License from Servier Medical Art (https://smart.servier.com/).

Many reviews have provided a detailed analysis of various flexible and wearable electrodes and sensors for electrophysiological measurements [17, 18, 19]. However, these reviews have generally paid limited attention to vertically aligned nanopillar electrodes as a distinct electrode class [17, 18, 19, 20, 21]. Therefore, this review provides a detailed and up‐to‐date summary of recent scientific advances in vertically aligned nanopillar electrodes for biophysical measurements (Figure 1). Section 2 explores various types of biophysical measurements, their importance, mechanism, development, performance evaluation, and their applications. In Section 3, we provide an overview of the types of vertically aligned electrodes and demonstrate the methods of recording the electrical activities in the human body by applying invasive, non‐invasive, and vertically aligned pillar‐like electrodes. In addition, Section 3 summarizes the key characteristics of vertically aligned electrodes including conductivity and high signal‐to‐noise ratio (SNR), adhesion, breathability, biocompatibility and biodegradability, flexibility and stretchability. In Section 4, we present several types of materials for fabricating vertically aligned nanopillar electrodes, including metal, carbon, and polymer‐based materials. Section 5 reviews various methodologies for the fabrication of vertically aligned nanowire or pillar‐like electrodes including lithography and template‐assisted techniques. Additionally, the applications of vertically aligned pillar electrodes for biophysical measurements are explained in Section 6, emphasizing the positive aspects of employing vertically aligned nanopillar electrodes for electrophysiological measurements. Finally, we present a conclusion, and we discuss the prospects for developed vertically aligned nanopillar electrodes.

## 2 Types of Electrophysiological Measurements

**FIGURE 2:**
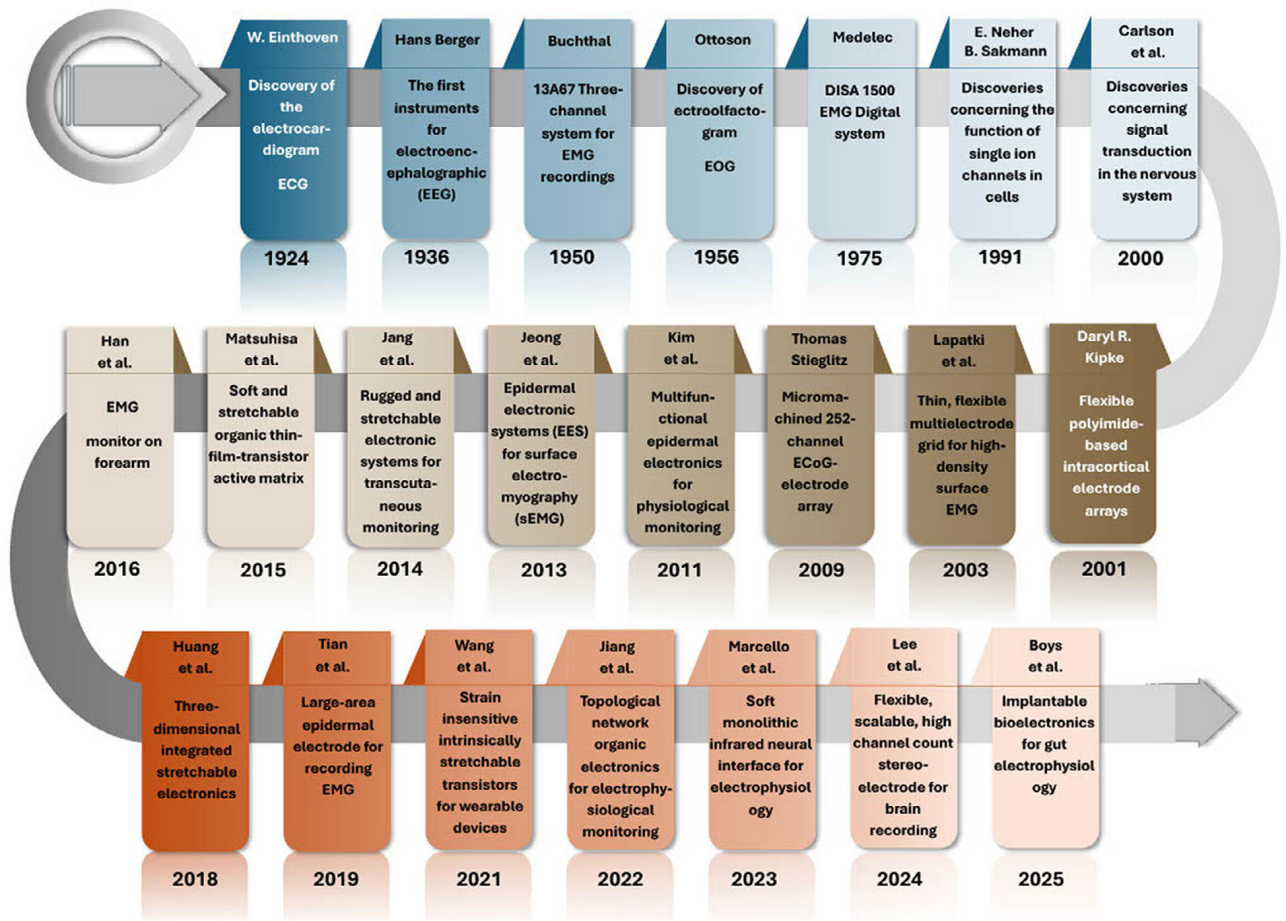
Chronological overview of major advancements in electrophysiological measurement technologies, from early ECG systems to modern flexible and stretchable bioelectronics [6, 22, 23, 24, 25, 26, 27, 28, 29, 30, 31, 32, 33, 34, 35, 36, 37, 38, 39, 40, 41, 42, 43].

Biophysical measurements play a central role in clinical diagnosis, physiological monitoring, and wearable healthcare technologies. Some of the most notable advances in this area are summarized in Figure 2. The initial discovery of the ability to non‐invasively read the beating signal of the heart in 1924 was the cornerstone in this journey and has since transformed many lives and benefitted countless patients.

**FIGURE 3:**
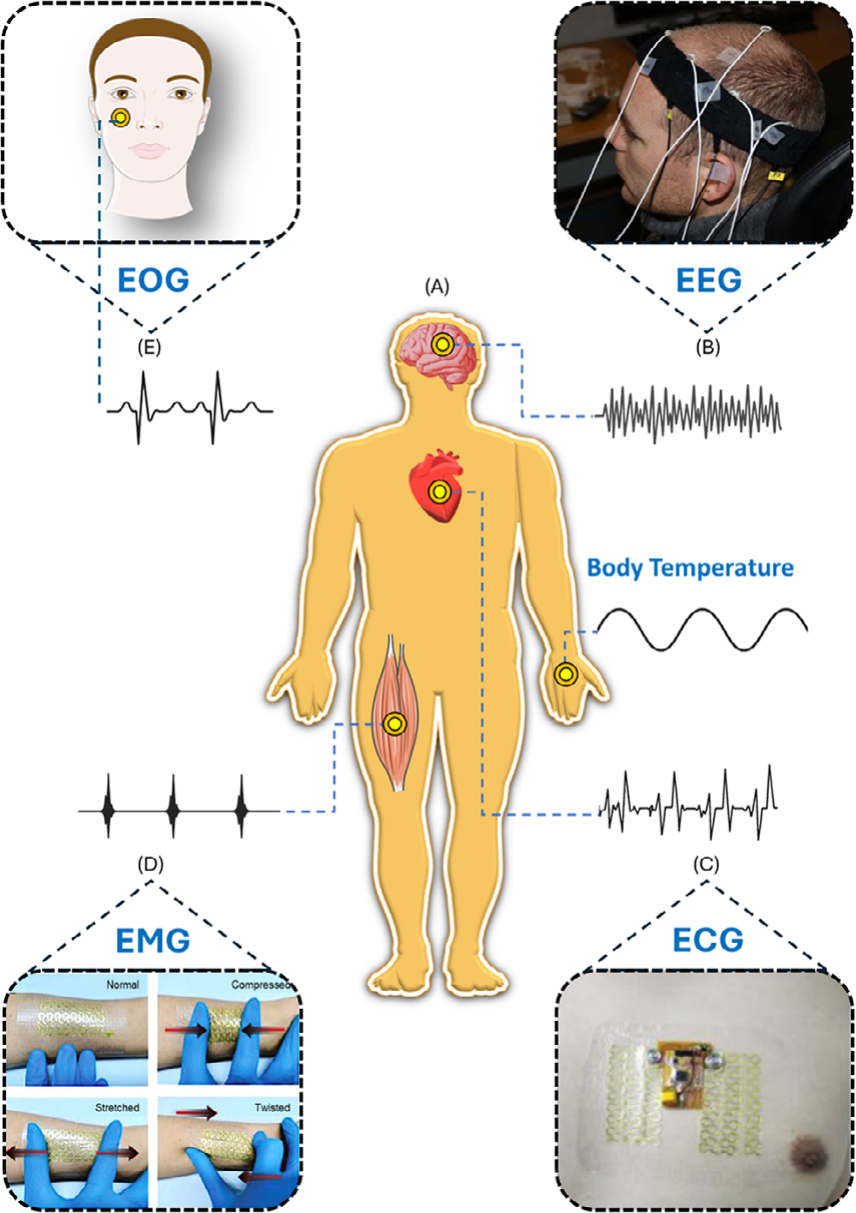
(A) Schematic of the biophysical measurements in the human nervous system and muscle system including EEG, ECG, EOG, EMG, and body temperature measurements. (B) EEG setup with textile and standard electrodes placed in close proximity using electrode gel. Reproduced with permission [52]. Copyright 2012, MDPI. (C) Thin flexible electrodes for ECG recording. Reproduced with permission [53]. Copyright 2021, Wiley‐VCH. (D) Shows the e‐tattoo electrode designed to capture EMG signals from the skin during normal, compression, stretching, and twisting. Reproduced with permission [53]. Copyright 2021, Wiley‐VCH. (E) Illustration of an EOG electrode for recording eye movement signals.

These tools are specified based on the electrophysiological parameters they measure for specific tissues/organs. Among the most widely used modalities are ECG [44], EEG [45], EMG [46], EOG [47], and temperature [48] (Figure 3), each providing important information about the functional state of major organs and physiological systems [49, 50, 51].

Although these modalities differ in signal origin, amplitude, and site acquisition, they share common interface requirements, including low contact impedance, reduced motion artifacts, conformal skin contact, long‐term comfort, and minimal irritation. These considerations are particularly important for continuous and wearable monitoring, where electrode performance depends strongly on interfacial design. Accordingly, understanding the relationship between biophysical signal requirements and electrode interface engineering is essential for evaluating the role of vertically aligned surface architectures in improving signal acquisition.

### 2.1 Electrocardiogram (ECG)

Cardiovascular diseases (CVDs) remain a major global health concern, increasing the demand for reliable heart‐rate and heartbeat monitoring technologies [54, 55]. Among the available techniques, ECG is widely used because it provides direct information on the electrical activity of the heart and supports the diagnosis, management, and prevention of CVDs [54, 55]. ECG monitoring is applied in hospitals, home settings, ambulatory systems, and telemonitoring platforms [56, 57, 58, 59, 60].

Typical ECG acquisition requires stable skin contact, low interfacial impedance, and resistance to motion‐induced noise, particularly for long‐term and wearable monitoring. Conventional ECG electrodes are commonly placed on the chest to detect the depolarization and repolarization of cardiac muscle during each heartbeat (Figure 3C) [53]. The electrocardiogram (ECG) signal exhibits a repetitive pattern consisting of a P‐wave, QRS complex, and T‐wave [61]. Because ECG signals are relatively weak, typically ranging from 10 µV to 4 mV, they are highly susceptible to interference and motion artifacts. Their standard acquisition bandwidth is 0.05–150 Hz, with most of the signal energy concentrated between 0.05 and 40 Hz [62]. These requirements highlight the importance of electrode interfaces that can maintain conformal, low‐noise, and irritation‐free contact with the skin, which provides the rationale for exploring vertically aligned electrode architectures.

### 2.2 Electroencephalography (EEG)

EEG is a well‐established, non‐invasive technique for monitoring brain activity and has become an essential tool in neuroscience and clinical neurology [63]. By recording voltage fluctuations generated by the coordinated firing of large neuronal populations through electrodes placed on the scalp, EEG provides insight into brain states, mental processes, and neurological disorders. EEG signals are typically weak, with amplitudes ranging from 5 to 300 µV and frequencies generally below 100 Hz [64]. It is widely used for the diagnosis and monitoring of conditions such as sleep disorders, movement disorders, and neurodevelopmental abnormalities.

EEG places stringent demands on electrode design because its low‐amplitude signals are highly sensitive to contact impedance, motion artifacts, and poor scalp conformity, particularly during long‐term monitoring. In this regard, recent advances in biogel‐based and textile electrodes have improved EEG recording quality by enhancing scalp contact and wearer comfort [52, 65]. Compared with other brain imaging techniques, EEG also offers high temporal resolution and portability, making it suitable for real‐world monitoring outside clinical settings. These requirements highlight the need for electrodes that interfaces with stable and low‐impedance, and create a comfortable contact with the scalp skin are important, providing a strong rationale for the use of vertically aligned surface architectures.

### 2.3 Electromyography (EMG)

EMG measures the electrical activity generated by muscles and is widely used to assess neuromuscular function in applications such as rehabilitation, physiotherapy, ergonomics, sports science, and movement disorder monitoring [66]. Surface EMG offers a non‐invasive approach by placing electrodes on the skin over the target muscle, whereas needle electrodes are used when deeper or more localized evaluation is required [67]. Because EMG is commonly performed on highly mobile body regions such as the face, arms, and legs, signal acquisition is particularly susceptible to motion artifacts, making electrode placement and interface stability critical [67]. EMG requires electrodes that can maintain stable skin contact, low motion‐induced noise, and long‐term wearing comfort, especially for continuous health monitoring [68]. Surface EMG signals typically exhibit amplitudes of approximately 0.1 µV to 5 mV and an effective frequency range of 10–500 Hz [69]. These features require electrode interfaces that can conform closely to the skin and preserve signal quality under mechanical deformation. As illustrated in Figure 3D. Stretchable e‐tattoo electrodes can maintain conformal contact under compression, stretching, and twisting, highlighting the importance of mechanically robust interfaces for wearable EMG monitoring [53]. These requirements provide a strong rationale for vertically aligned electrode structures that can improve interfacial stability and reduce motion‐related signal degradation.

### 2.4 Electrooculography (EOG)

EOG is a non‐invasive technique used to record the electrical potentials generated by eye movements and has been applied in areas such as clinical ophthalmology and human‐computer interaction [70, 71]. By placing electrodes around the eyes (Figure 3E), EOG captures changes in the electrical field produced as the cornea moves relative to the retina, enabling the detection of eye motion and blinking activity [71, 72].

EOG also requires electrodes that can maintain stable, low‐noise contact on delicate and highly mobile periorbital skin. However, key differentiator originates because of the electrode placement area, which is sensitive and repeatedly exposed to blinking and facial motion, minimizing motion artifacts, discomfort, and skin irritation is particularly important for reliable signal acquisition. These demands make soft, conformal, and mechanically stable electrode interfaces especially desirable, providing a clear rationale for vertically aligned surface structures that can enhance contact stability while preserving user comfort.

### 2.5 Temperature Measurement

Skin temperature is an important physiological indicator, as deviations from the normal body temperature range may reflect disease or abnormal health conditions [73]. Normal human body temperature is typically around 37°C–37.5°C [74], and even small fluctuations can be clinically meaningful, requiring sensors with high sensitivity for accurate monitoring [75].

Usually, temperature sensing requires intimate, stable, and irritation‐free contact with the skin to ensure accurate and continuous signal acquisition. Conventional rigid thermometers can create local pressure points and are not well suited for continuous or wearable monitoring, particularly in confined environments or for infants and young children [74, 76]. These limitations have driven the development of flexible and wearable temperature sensors that are soft, lightweight, biocompatible, durable, and conformal to the skin [77, 78]. Therefore, maintaining close interfacial contact is a key design requirement, which supports the use of structured surfaces and vertically aligned architectures to improve contact stability and wearer comfort. Resistance temperature detectors (RTDs), thermocouples, and thermistors remain the main sensor types used for temperature monitoring [79].

Collectively, these biophysical monitoring modalities impose common requirements on the electrode‐skin interface, including low contact impedance, reduced motion artifacts, conformal contact, long‐term comfort, and minimal skin irritation. These constraints are particularly important for continuous and wearable applications, where signal stability depends strongly on interfacial design. Accordingly, vertically aligned surface architectures offer distinct advantages by increasing effective contact area, improving mechanical interlocking, and enhancing interfacial stability, thereby supporting more reliable biophysical signal acquisition.

## 3 Types of Vertically Aligned Nanopillar Electrodes and Their Characteristics

Nanostructured electrodes have attracted considerable attention because their micro‐/nanotextured architectures can significantly improve interfacial performance. Compared with flat surfaces, vertically aligned nanoarchitectures provide a much larger effective surface area, thereby increasing the functional surface area of the electrode without changing its overall dimensions [80]. Hierarchical micro/nanoscale structures, such as pillar arrays, have been engineered to exploit this advantage. Nanopillars can enhance biosensing performance by increasing surface area, which may amplify the detected signal, improve SNR, and consequently enhance sensitivity [81]. Table 1 summarizes representative nanopillar electrodes and compares their key performance parameters, including material type, impedance, SNR, adhesion, stability, and compatibility.

**TABLE 1:** Comparison of representative nanopillar electrodes including material, impedance, SNR, adhesion, stability, and compatibility.

Material	Signal / SNR	Adhesion	Stability	Biocompatibility / compatibility	Refs.
Pt nanopillars	100–200 µV SNR 4.5–9 intracellular 11.8 mV SNR 590	Strong membrane coupling	Minutes to days	HL‐1 compatible	[20]
Quartz nanopillars	Up to 60 cells SNR 838	Stable intracellular access	Drug‐screening capable	hPSC‐CM compatible	[82]
Au nanopillars	0.8–3 mV	Enhanced coupling	>8 repeats over >10 days	Compatible	[83]
CMOS nanopillars	∼5 mV	Network‐level coupling	Scalable recording	Compatible	[84]
Plasmonic nanopillars	100–400 µV SNR 4.5	Good sealing	Up to 80 min	Neuron/HL‐1 compatible	[85]
Si nanopillars	Good SNR	Not reported	Stable	Compatible	[86]
Si nanowires	SNR >1000	Improved coupling	∼30 min	Compatible	[87]
Fused‐silica nanopillar electrode	Not reported	Intracellular‐like neuron coupling	Several hundred seconds	Compatible	[88]
Au nanopillar array	N/A	Not reported	Electrochemical response stable enough for glucose sensing	glucose oxidase functionalized biosensor surface	[89]
Flexible Au/Ag nanopillar‐array electrode	N/A	Not reported	Reproducibility <10%	Insulin sensing in plasma samples	[90]

In addition to their surface‐area advantage, vertically aligned nanopillars can improve cell‐electrode interactions through their distinctive morphology. Their ordered arrangement promotes cellular positioning between adjacent pillars and can enhance cellular adhesion, which is beneficial for biointerfacing applications, including implantable and diagnostic devices [91]. In some cases, tapered or pointed nanopillar geometries can also facilitate localized membrane interaction, enabling delivery of biomolecules or intracellular access depending on the device design and application [92].

**FIGURE 4:**
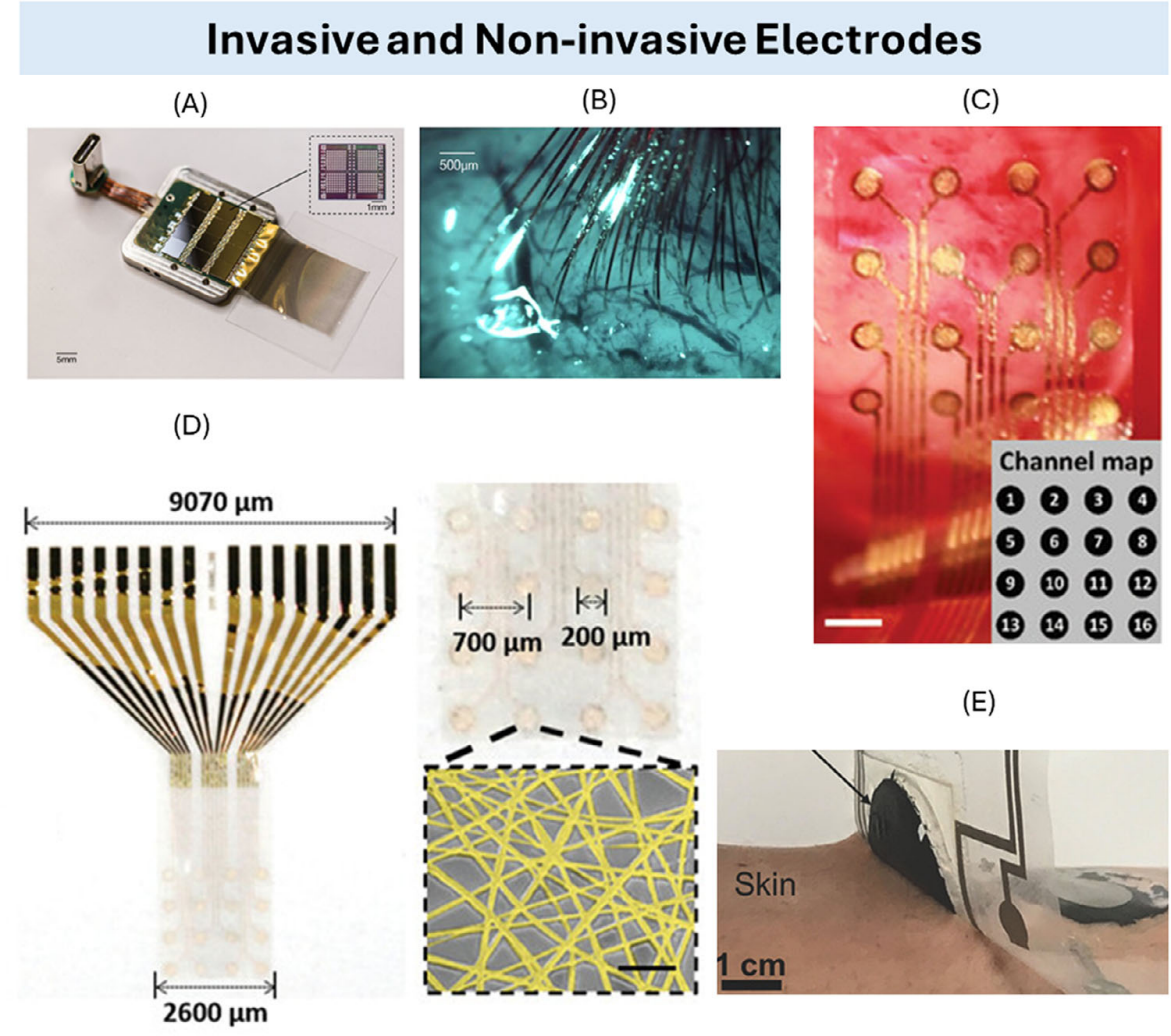
Shows different forms of invasive and non‐invasive electrodes. (A) A packaged sensor device contains 12 chips for a total of 3072 channels. Reproduced with permission [95]. Copyright 2019, JMIR. (B) Image showing the cortical surface with implanted threads in a rat cortex. Reproduced with permission [95]. Copyright 2019, JMIR. (C) Placement of the Au NN ECoG microelectrode array onto the cerebral cortex via a cranial window. Reproduced with permission [96]. Copyright 2020, WILEY‐VCH. (D) Optical images of the Au NN ECoG array display 16 recording channels, each consisting of circular electrodes with a diameter of 200 µm and spaced 700 µm apart. Reproduced with permission [96]. Copyright 2020, WILEY‐VCH. The overall device width is 2.6 mm. The magnified view at the bottom right shows a SEM image of the Au NN electrodes, highlighted in yellow. Scale bar: 5 µm. (E) Photo of the adhesion test of the ECG device peeled from skin. Reproduced with permission [97]. Copyright 2017, WILEY‐VCH.

According to their mode of operation, electrodes are often broadly categorized as invasive and non‐invasive [18]. However, this classification is simplified, as some emerging electrode interfaces may occupy an intermediate regime depending on their geometry and mode of interaction with tissue. Invasive electrodes generally provide higher SNRs and improved accuracy [93], but their placement may require surgical intervention, which increases health and infection risks. In contrast, non‐invasive electrodes are generally safer and less disruptive to the patient [94], although they are often more susceptible to environmental interference, reduced consistency, and the need for frequent calibration [94]. Therefore, the choice between these electrode types depends on the specific demands of the intended application. Vertically aligned nanopillar electrodes have been explored in both invasive and non‐invasive settings for improved biophysical monitoring, as shown in Figure 4.

### 3.1 Invasive Electrodes

Typically, invasive electrodes are surgically implanted into specific regions of the body, such as the brain, spinal cord, heart, and limb muscles. These electrodes enable signal acquisition with higher accuracy and are primarily employed in patients for whom non‐invasive approaches are ineffective or insufficient for accurate diagnosis [98]. Invasive electrodes offer two distinctive features: Initially, they can sense the voltage signals from a particular area around the electrodes, typically located deeply instead of the surface of the investigated area [99]. Second, they have the ability to focus on specific neurons at specific locations allowing for real‐time detection of electrical signals. These distinct abilities have resulted in effective methods for interpreting and improving the human health tracking system. Typically, invasive electrodes generally are the electrodes placed onto various regions of the body, including the brain, spinal cord, heart, and limb muscles via surgical implantation.

A major application of invasive electrodes lies in neural interfacing for human‐machine interaction, where neurological signals are acquired and transduced into control commands for external devices. Advancements in brain‐machine interface (BMI) technology continue to show promise for the recovery of motor and sensory capabilities. Neuralink has developed a high‐resolution BMI platform (Figure 4A) that uses flexible threads (Figure 4B) to deploy up to 3072 electrodes, with an 85.5% spiking yield observed in long‐term implants [95]. Developments in transparent neural interfaces have made it possible to perform electrophysiological recordings alongside optical stimulation without introducing photoelectric interference. Arrays based on gold nanonetworks (Au NNs) (Figure 4D) offer high optical transmittance (81%) and low impedance (33.9 kΩ at 1 kHz), enabling high‐resolution, artifact‐free 2D mapping (Figure 4C) of neural activity during optogenetic stimulation [96].

Most commonly applied invasive electrodes consist of two primary types: thin‐film microelectrode arrays and 3D invasive electrodes [100]. Si‐based Integrated circuits could make it possible to create high‐density arrays of intracellular electrodes, which would allow for the measurement of hundreds of cells [101]. Coaxial silicon nanowires with atomic gold surfaces can effectively stimulate neurons via photoelectrochemical mechanisms [102]. TiO2‐Au nanowire motors can accurately target and stimulate retinal ganglion cells through electric fields created by UV light [103]. The primary challenge for invasive electrodes may damage tissues, harm cells, and initiate foreign body consequences, perhaps leading to a significant decrease in the amplitude of the acquired signals.

### 3.2 Non‐Invasive Electrodes

Non‐invasive electrodes are designed to capture electrical signals without penetrating the skin. In contrast to invasive approaches, which require complex surgical implantation, on‐skin electrodes offer a far more practical and user‐friendly alternative for routine use. Their ease of application greatly improves accessibility and expands their potential for widespread adoption in personal health monitoring and home‐based healthcare.

Recent developments in vertically aligned nanopillar electrodes have demonstrated the ability to perform non‐invasive electrophysiological assessments. Silicon nanowire arrays produced by complementary metal oxide semiconductor (CMOS) compatible methods allow non‐invasive real‐time extracellular recording of cellular bioelectricity with high sensitivity and throughput [104]. For wearable applications, silver nanowire dry electrodes were attached to the wrist for ECG measurements, or to the forearm for EMG measurements [105]. These electrodes have shown comparable performance to traditional Ag/AgCl wet electrodes for ECG and EMG with reduced motion artifacts and no skin irritation. In addition, researchers have developed effective reliable gel‐free ECG electrodes (Figure 4E), optimizing film thickness for accurate measurements [97]. A temporary tattoo electrode composed of metal was introduced by Rogers et al., featuring ultra‐thin electrodes and interconnects just a few microns thick fabricated on a polyvinyl alcohol (PVA) sacrificial layer [106]. Upon transferring the complete device onto the skin, the PVA layer was dissolved, allowing the electrode to adhere conformally to the skin via van der Waals forces. This conformal attachment enabled reliable recording of electrophysiological signals.

In addition, vertically aligned nanopillar arrays can efficiently pin the position of neurons in a cultured neural network without negatively affecting neuronal growth and development, facilitating long‐term observation of the same neurons [107]. These advances highlight the considerable potential of non‐invasive nanopillar electrodes across a wide range of biomedical applications. However, a major challenge for non‐invasive thin‐film nanopillar electrode arrays lies in achieving reliable conformal attachment to the complex 3D surfaces of organs. It is essential to preserve signal stability during organ deformation associated with physiological activity while simultaneously minimizing the risk of tissue damage.

### 3.3 Vertically Aligned Nanopillar Electrodes Characteristics

**FIGURE 5:**
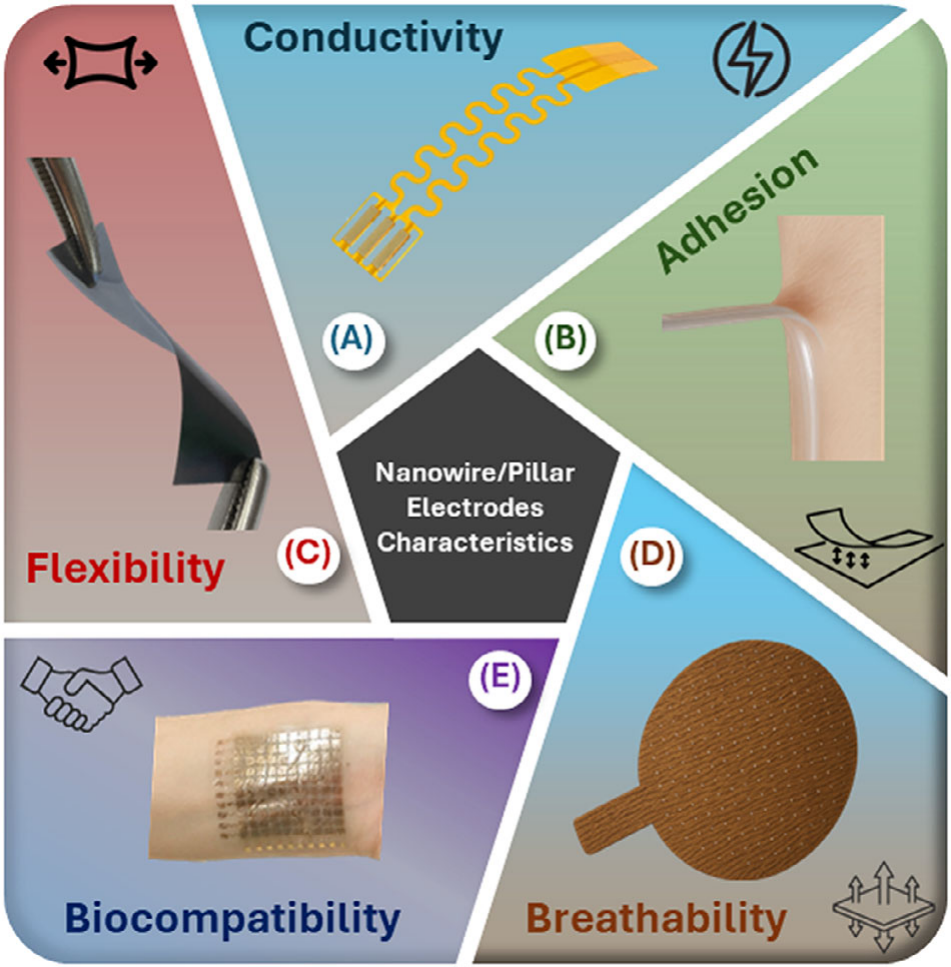
Illustration of the key characteristics for high‐performance pillar electrodes. (A) Reproduced with permission [108]. Copyright 2020, Nature. (B) Reproduced with permission [109]. Copyright 2023, Nature. (C) Reproduced with permission: Copyright 2016, American Chemical Society [110]. (D) Reproduced with permission [111]. Copyright 2022, Frontiers. (E) Reproduced with permission [112]. Copyright 2017, Elsevier.

Vertically aligned nanopillar electrodes have emerged as advanced architectures for bioelectronic interfaces due to their unique structural and electrical properties. Their nanoscale geometry, characterized by high aspect ratios and enlarged surface areas, promotes enhanced cell‐electrode interactions, efficient electrical signal transduction, and improved spatial resolution. These features make them particularly suitable for applications such as neural recording, stimulation, and intracellular sensing. Furthermore, their compatibility with flexible substrates enables the development of next‐generation wearable and implantable bioelectronic devices. Figure 5 illustrates the key features of high‐performance nanopillar electrodes.

#### 3.3.1 Conductivity and High Signal‐to‐Noise Ratio (SNR)

Electrode materials are required to obtain high conductivity and low impedance to accurately capture electrophysiological signals with a high SNR. High conductivity enhances the SNR of electrophysiological signals by creating a strong surface electric displacement field and charge carrier density.

Nanopillar electrodes have shown promising conductivity and stretchability for flexible electronics applications. The researchers built a highly flexible and conductive metal electrode by a solution‐processing technique, forming a percolation network of very long metal nanowires [113]. Metals such as Ag/AgCl exhibit high conductivity features resulting in exploiting them in the fabrication of electrodes. The scientists employed a plasmon‐enhanced nanosoldering (PLNS) technique to improve the conductivity of silver nanowire (Ag NW) electrodes by locally soldering the Ag nanoparticles (NPs) that make up the Ag NWs [114].

In silicon nanowire field‐effect transistors, the SNR is optimized at maximal transconductance and is mainly determined by the intrinsic quality of the device rather than the characteristics of the electrolyte [115]. A recent model indicates that the design of nanopillar arrays and the state of cell adhesion considerably influence the SNR, with the best performance attained by reducing nanopillar spacing and height while enhancing radius [116]. These findings emphasize the significance of nanopillar electrodes in improving conductivity and SNR for sensitive applications.

#### 3.3.2 Adhesion

Electrodes offer numerous advantages that support their use across a broad range of applications, including biophysical, electronics, medical measurements, therapy, and scientific research. Among these advantages, adhesion is particularly important. Effective adhesion to human skin is essential for maintaining stable, long‐term biophysical monitoring. In this regard, vertically aligned nanopillar electrodes have demonstrated significant potential for improving adhesion performance. Certain cell types, including heart muscle and kidney cells, exhibited strong adherence to gold nanopillar electrodes [117]. Recent studies have focused on enhance the adhesion of silver nanowire (AgNW) electrodes to substrates, tackling a significant obstacle in the advancement of flexible transparent electrodes [118]. Fischer et al. illustrated the efficacy of nanowire coatings in biomedical applications, considerably improving adherence in mucosal conditions for drug delivery systems [119].

#### 3.3.3 Flexibility and Stretchability

Biophysical monitoring devices may be susceptible to failure or performance degradation during use, particularly in health and fitness applications where sweating or exposure to aqueous environments is common. To improve patient comfort and convenience during signal recording, advanced electronic systems have been developed with properties such as water resistance, breathability, and mechanical flexibility. Consequently, the development of biophysical monitoring platforms with flexible and stretchable characteristics has attracted substantial global interest [120].

Flexibility refers to the capability of electrodes to sustain their conductivity when subjected to mechanical strain [121]. Vertically aligned nanopillar electrodes provide enhanced flexibility and stretchability for many applications in flexible electronics. These features enhance adhesion and strain distribution, allowing excellent stretchability while maintaining conductivity [122]. Polyoxometalate‐doped polypyrrole nanopillar arrays offer improved electrochemical performance and mechanical qualities, enabling bending and twisting without loss of functionality [110]. These advancements in nanopillar electrode technology support applications in wearable electronics, biointegrated devices, and advanced flexible displays.

Although vertically aligned nanopillar electrodes are often highlighted for their flexibility and intimate tissue coupling, their mechanical durability under lateral or shear loading remains a critical concern [123, 124]. High‐aspect‐ratio nanostructures are inherently susceptible to collapse, bending, and fracture when geometric stability is insufficient, and this concern becomes more significant during repeated motion, prolonged wear, or implantation‐related micromotion [123, 124]. In addition, the long‐term performance of these biointerfaces depends not only on electrical function but also on preserving structural integrity at the cell/material interface [124]. If fractured nanostructure fragments are released into surrounding tissue, they may further aggravate the foreign body response and inflammation [125], while implant‐derived nano/submicron debris has also been linked to cytotoxic and pro‐inflammatory effects [126]. Therefore, future electrode designs should consider not only flexibility, but also fracture resistance, anchoring strength, and debris‐related biocompatibility.

#### 3.3.4 Breathability

While the majority of skin bioelectronics on the market today use a film‐type structure with minimal permeability, in a healthy microenvironment, the skin is able to allow air, steam, and liquid to pass through [127]. Due to gas permeability and lack of inflammatory properties, breathability enhances the biocompatibility of biophysical sensors [128]. Breathability is a critical parameter for ensuring user comfort and minimizing skin irritation in applications involving biophysical electrodes and wearable sensors. Improved breathability facilitates effective dissipation of heat and moisture, thus enhancing overall user experience and long‐term wearability.

Recent research efforts have concentrated on designing breathable and flexible electrodes for wearable electronics. These electrodes often consist of silver nanowires (AgNWs) integrated into polymer nanofiber scaffolds, providing excellent conductivity and optical transmittance [129]. Moreover, dry electrodes created by semi‐embedding AgNWs onto modified polyvinyl alcohol have demonstrated significant stretchability, air permeability, and adhesion, enabling stable electrophysiological signal monitoring during physical activity [130]. These improvements in nanowire/pillar‐based electrodes provide potential solutions for breathable electronic devices across numerous applications, including health monitoring and human‐machine interfaces.

#### 3.3.5 Biocompatibility

In addition to conductivity, adhesion, and breathability, biocompatibility is another critical consideration in the design of biophysical electrodes. To minimize the risk of adverse effects, such as skin irritation or allergic reactions, the materials used in these electrodes must be biocompatible. The use of biocompatible materials ensures safe long‐term contact with the skin without causing tissue damage or discomfort [131]. Zinc oxide ZnO nanowires demonstrate solubility in biofluids, suggesting possible biodegradability and allowing them to be used for in vivo biosensing and biodetection applications [132]. Silicon nanowires (SiNWs) exhibit biocompatibility as substrates for cell growth, enabling high‐resolution bioelectric signal recording through CMOS‐compatible manufacturing [133]. Furthermore, Conductive biopolymer nanocomposites incorporating poly(3‐hydroxybutyrate) and nanowires have been effectively evaluated as flexible, biocompatible electrodes for transcutaneous electrical nerve stimulation and ECG [122]. These investigations demonstrate the promise of diverse nanopillar electrode materials and designs for biomedical applications, addressing concerns of biocompatibility and biodegradability.

## 4 Materials for Vertically Aligned Nanopillar‐Like Electrodes

**FIGURE 6:**
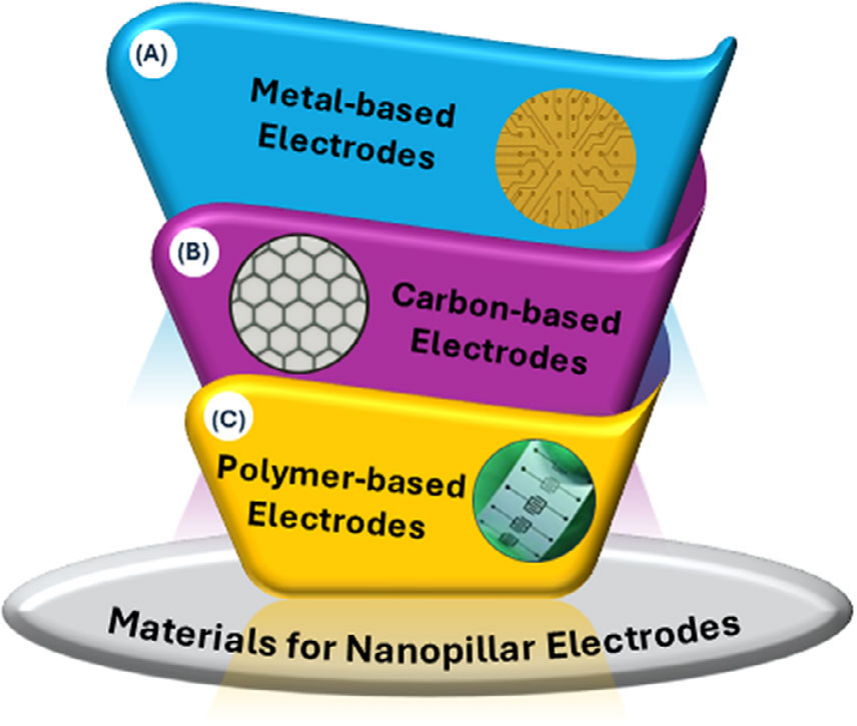
A schematic to demonstrate the nanopillar electrodes base materials including (A) metal, (B) carbon, and (C) polymer. Reproduced with permission [142]. Copyright 2011, WILEY‐VCH.

Vertically aligned nanopillars composed of non‐toxic, corrosion‐resistant metals have attracted considerable attention across a range of applications owing to their high aspect ratios and favorable physicochemical properties. Nanowire and nanopillar electrodes can be fabricated from diverse material classes, including metal‐, carbon‐, and polymer‐based systems, each offering distinct advantages depending on the intended application, as illustrated in Figure 6. Common materials exploited for fabricating vertically aligned nanopillars electrodes are gold (Au) [134], silver (Ag/AgCl) [135], stainless steel [136], copper (Cu) [137], zinc (Zn) [138], titanium (Ti) [139], silicon (Si) [140], and platinum (Pt) [141] due to their distinctive features.

### 4.1 Metal‐Based Vertically Aligned Nanopillar Electrodes

**FIGURE 7:**
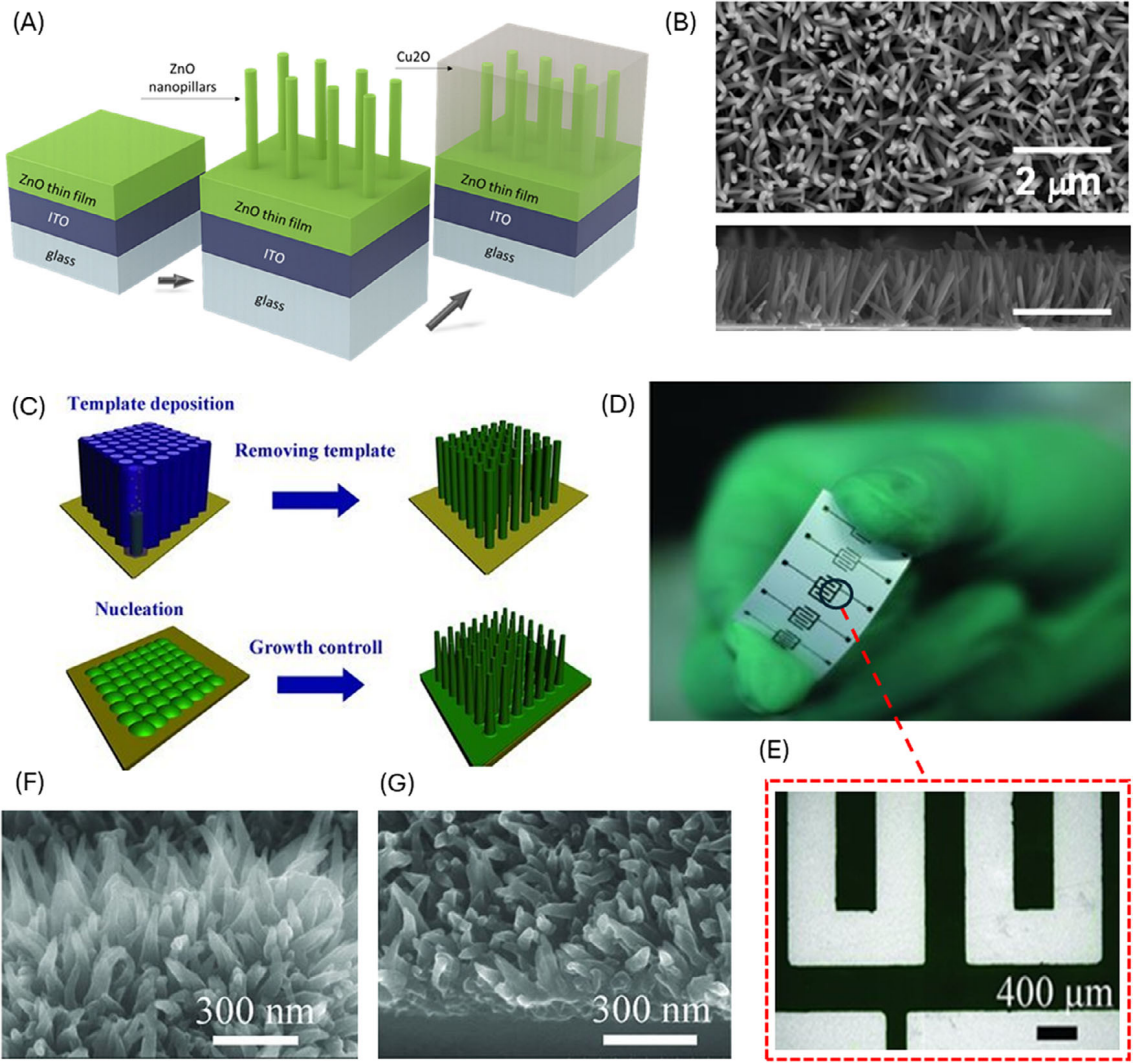
Overview of various vertically aligned nanopillar electrodes categorized by material type. (A) Fabrication process of Cu_2_O/ZnO nanopillar heterostructured electrodes. Reproduced with permission [156]. Copyright 2010, ACS. (B) SEM images of top view and cross section of ZnO nanopillars. Reproduced with permission [156]. Copyright 2010, ACS. (C) A schematic of the two common approaches for fabricating conducting polymer nanowire arrays: (top) the template‐assisted method and (bottom) the template‐free method. Reproduced with permission [160]. Copyright 2013, WILEY‐VCH. (D) An optical image showing flexible micro‐supercapacitor unit arrays fabricated on a PET substrate. Reproduced with permission: Copyright 2011, WILEY‐VCH [142]. (E) Optical image of MSC 400; SEM images of PANI nanowire arrays captured from (F) a 20° tilted angle and (G) a top view. Reproduced with permission [142]. Copyright 2011, WILEY‐VCH.

Metal‐based electrodes have been previously employed for a variety of purposes, such as recording electrical signals and stimulation (Figure 7). They have been utilized due to their excellent conductivity and durability. In addition, metal‐based electrodes have been significantly exploited in enhancing and understanding electrical activities of human. Metal‐based electrodes have numerous benefits and advantages for human health monitoring. Their considerable conductivity facilitates excellent signal transmission between the electrode and human tissues, leading to precise and reliable recordings. considerable conductivity facilitates excellent signal transmission between the electrode and human tissues, leading to precise and reliable recordings. A broader comparison of representative metal‐, carbon‐, and polymer‐based nanopillar electrodes is presented in Table 2.

**TABLE 2:** Summary of representative metal‐, carbon‐, and polymer‐based nanopillar electrodes.

Electrode material	Fabrication method	Conductivity/resistivity	SNR/amplitude/impedance	Refs.
Metal‐based vertically aligned nanopillar electrodes				
Ag nanowire	Spin coating	≈ 47.4 Ω/sq	High amplitude (peak at ∼400 Hz) High SNR (∼98.6%),	[143]
Au nanopillar	Template‐assisted PAA	—	Higher amplitude 1.50 ± 0.02 mV RMS noise 6.6 µV	[144]
Cu‐Au nanowire	Hydrothermal method	100‐200 Ω	SNR 18.05	[145]
Pt nanopillar	Electroporation	—	Amplitude: 11.8 mV SNR: 590	[20]
Ag nanowire	Selective‐patterning coating	1.52–4.35 Ω/sq	Impedance 3.78–6.04 Ω·cm^2^	[146]

Carbon‐based vertically aligned nanopillar electrodes				
VACNF arrays	Vertically aligned carbon nanofiber growth on microelectrodes	—	Impedance 4–7 MΩ at 1 kHz amplitude ∼360 µV SNR ∼8:1	[147]
Suspended carbon nanowire	SU‐8 lithography	—	120× current amplification	[148]
CNF microelectrode	Electrochemical deposition	—	Impedance = 1.28 MΩ at 1 kHz	[149]

Polymer‐based vertically aligned nanopillar electrodes				
HP‐AgNW/TPU	Breath figure method	7.9 Ωsq^−1^ transmittance 61%	SNR 24.9	[150]
PEDOT:PSS/AgNW/PEDOT:PSS	Spin coating	—	Impedance = 50 KΩ (100Hz)	[151]
HPAN/PU/AgNW	Electrospinning, vacuum filtration	—	SNR 25	[152]
PEDOT:PSS‐coated Au nanopillars	Template‐based electrodeposition	—	Impedance >350× smaller compared to planar gold electrodes at 1 kHz	[153]
PEDOT:PSS microwires	Electrochemical synthesis	Conductivity ∼30 S/cm diameter 860 nm‐4.5 µm	low Young's modulus (∼1 GPa)	[154]
Au‐ZnO‐Au‐PEDOT nanowire neural electrode	Electrodeposition	—	Impedance improvement ∼35× at 1 kHz and ∼105× at 200 Hz vs comparison structure	[155]

A simple two‐step electrodeposition method (Figure 7A) was used to fabricate Cu2O/ZnO nanopillar solar cells (Figure 7B) with improved efficiency compared to planar thin film junctions [156]. Moreover, it has also been proven that nanopillar electrodes have been made using silver (Ag) [135]. These Ag nanopillar‐like electrodes, known for their increased sensitivity, excellent mechanical stability, and great surface area, are considered to be one of the most promising substitutes for flat electrodes. Ag especially in the form of nanowires and ink deposition, has become popular because it allows for different production techniques and can be embedded in flexible polymer substrates to enhance flexibility [157].

Controlled radiofrequency RF plasma processing can fabricate nanopillar structures on biomedical alloys, such as stainless steel, therefore decreasing electrode impedance for enhanced performance [158]. Carbon nanotubes developed on stainless steel plates and wires serve as efficient electrodes for chemical and biological sensing, exhibiting favorable redox behavior and radial diffusion characteristics [159].

Copper is widely recognized as a soft and ductile metal with excellent thermal and electrical conductivity. Owing to these properties, copper‐based materials have been extensively investigated for their potential use in flexible electrode applications. Oxidation‐resistant copper nanowires can be used to fabricate flexible, foldable, and free‐standing electrodes [161]. Anodization of copper in alkaline electrolytes, including sodium bicarbonate, sodium carbonate, and sodium hydroxide, can yield diverse nanostructures, such as nanowires, nanorods, and nanobundles [162, 163, 164]. The morphology and composition of these nanostructures are affected by variables involving the applied voltage, electrolyte concentration, and temperature [165]. Nanowires synthesized in sodium bicarbonate consist of Cu2O, CuO, Cu(OH)2, and malachite, exhibiting sizes between 35 and 45 nm [162].

Owing to its low corrosion susceptibility and lightweight nature, titanium has emerged as an attractive biocompatible metal and is widely used in therapeutic implants [166]. Titania vertically aligned nanopillars on titanium surfaces significantly affected human skeletal stem cell behavior, with 15 nm height features generating the most significant cellular response and bone matrix synthesis [167].

There have been considerable efforts to use titanium‐based nanopillar electrodes. Titania vertically aligned nanopillars on titanium surfaces significantly affected human skeletal stem cell behavior, with 15 nm height features providing the most significant cellular response and bone matrix synthesis [168]. Titanium nitride TiN nanowires demonstrate enhanced electrochemical stability and biocompatibility in comparison with thin films, exhibiting exceptional capacitance retention and consistent impedance throughout numerous cycles [169].

Platinum vertically aligned nanopillar electrodes created by Cheng et al. exhibit considerable enhancements in electrochemical performance compared to traditional flat electrodes [141]. Their work showed improved hydrogen evolution reaction (HER) efficiency with Pt nanopillar‐array electrodes, with a low overpotential of 78 mV at 10 mA cm^−2^. This enhancement was ascribed to reduced charge transfer resistance and enhanced hydrogen bubble desorption at nanotips. Despite these advantageous properties, their application as electrodes for biophysical measurements remains relatively limited, and key studies are discussed in detail in Section 6. Notably, vertically aligned gold nanopillars have been successfully fabricated on gold electrodes via template‐assisted electrode position, demonstrating significant potential due to their increased surface area, reduced impedance, and favorable compatibility with neural cells [134, 135]. Vertical gold nanopillars exhibit flexibility and retain superior electrochemical performance under tension, rendering them appropriate for wearable or implanted electronic devices [170].

Silicon vertically aligned nanopillar electrodes have demonstrated potential across multiple applications. High‐aspect‐ratio structures can be produced using metal‐assisted etching, deep UV lithography, and reactive ion etching, with diameters ranging from 10 to 70 nm and heights between 0.4 and 0.6 µm [171].

### 4.2 Carbon‐Based Vertically Aligned Nanopillar Electrodes

Carbon materials exhibit great electrical conductivity, flexibility features, and corrosion resistance, therefore, they are currently significant for electrode materials. Carbon nanotube (CNT) was fabricated in 1991 by Sumio Iijimaare [172]. Carbon‐based nanopillar electrodes have emerged as promising platforms for electrochemical sensing and neurotransmitter detection. Carbon‐based nanopillar electrodes have developed into potential platforms for electrochemical sensing and neurotransmitter detection. These nanostructures provide higher sensitivity with suspended carbon nanowire meshes obtaining 120‐fold signal amplification [148]. Single carbon nanotube electrodes offer superior spatial resolution and SNR in comparison to conventional carbon fiber probes [173]. Carbon‐based nanomaterials, including graphene and carbon nanotubes, facilitate the development of soft neural electrodes that exhibit lower inflammatory responses and sustained chronic in vivo recordings [174]. Carbon‐based nanopillar electrodes can be optimized for neurotransmitter detection through chemical and 3D surface structure modifications [175].

Various graphene‐based measurements have been developed for human health monitoring, such as wearable and implantable medical equipment that can provide real‐time assessment of various physiological parameters including heart rate, temperature of the body, blood oxygen levels, breathing rate, pressure in the blood, blood sugar, ECG, EMG, and EEG signals [176]. Graphene possesses exceptional properties including ultrahigh carrier movement, effective electrical and thermal conductivity, considerable surface area, elevated optical transmittance, increased Young's modulus, and exceptional mechanical flexibility. A scalable method for fabricating suspended carbon nanowire mesh electrodes was developed by Heo et al. using carbon micro electro mechanical systems MEMS technology, resulting in a remarkable 120‐fold enhancement in signal amplification [148] Researchers have also investigated graphene nanowires and pillared architectures for supercapacitor electrodes, exhibiting improved performance. Graphene oxide/polypyrrole nanowires demonstrated a high capacitance of 960 F g(‐1), whereas decreased graphene oxide/carbon nanowires showed enhanced thermal stability and long‐term charge storage [177].

### 4.3 Polymer‐Based Vertically Aligned Nanopillar Electrodes

Conducting polymers can be tailored through chemical functionalization, integration of nanostructures, or incorporation with other materials such as nanoparticles. These modifications can enhance key performance parameters, including sensitivity, selectivity, stability, and signal transduction capabilities [178]. Conducting polymer nanopillars can be synthesized via electrodeposition and mechanical stretching, demonstrating incremental conductance variations and adjustable current‐voltage (*I–V*) characteristics [179]. These nanostructures, especially aligned nanopillars arrays, show improved capacitance and exceptional rate capability, making them optimal for high‐performance supercapacitors [160]. Flexible micro‐supercapacitors (MSCs) integrated on chips can be combined with sensors and energy harvesting units to develop fully self‐powered electronic systems [142]. Figure 7C presents simplified schematic illustrations of the fabrication process for PANI nanowire arrays integrated into the MSC. Figure 7D shows a real image of the flexible micro‐supercapacitor unit arrays; (Figure 7E) optical microscope view of MSC 400. The microelectrode consists of vertically aligned polyaniline (PANI) nanowires as shown in Figure 7F (top view) and Figure 7G (tilted 20° view).

Moreover, individually addressable conducting polymer nanopillar arrays can be fabricated by electrodeposition between electrodes within predefined channels on semiconducting or insulating substrates, enabling precise control over their dimensions and spatial placement [180]. Such fabrication strategies allow the production of nanopillar structures with efficient electrical contact to the electrodes, making them well suited for a wide range of device and sensing applications.

## 5 Fabrication of Vertically Aligned Nanopillar Electrodes

**FIGURE 8:**
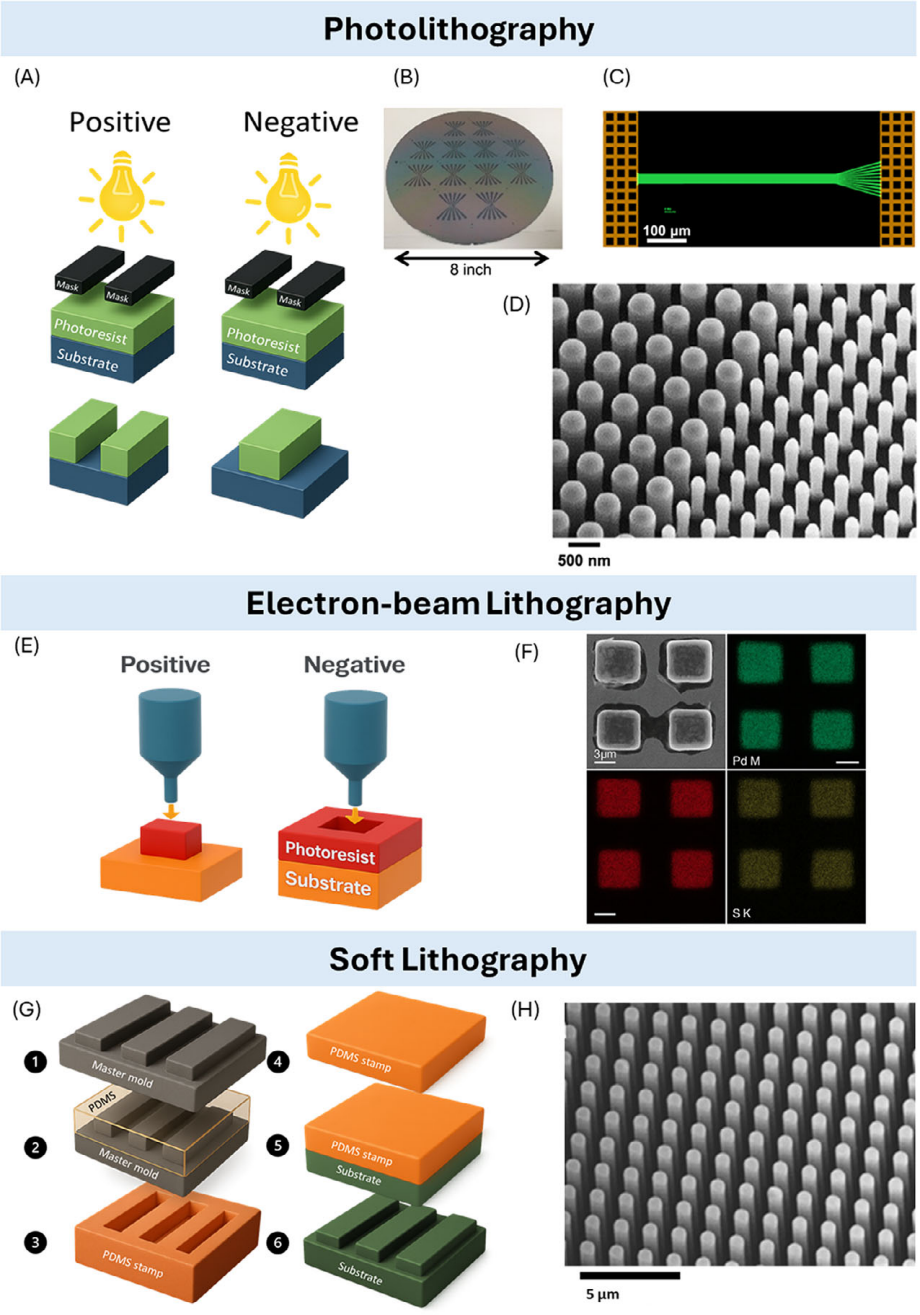
Illustrates various methods of creating patterns. (A) Photolithography schematic includes both positive and negative techniques. (B) Digital photograph of a 200 mm wafer featuring vertically aligned nanopillar array fabrication through the integration of electron beam lithography (EBL) and photolithography patterns. Reproduced with permission [208]. Copyright 2020, IOP Publishing. (C) Schematic layout illustrating the region where EBL and photolithography patterns are combined. Reproduced with permission [208]. Copyright 2020, IOP Publishing. (D) Tilted SEM image illustrating that the vertically aligned nanopillar array is composed of pillars with diameters of 450 nm (left) and 220 nm (right). Reproduced with permission [208]. Copyright 2020, IOP Publishing. (E) A schematic of E‐beam lithography with both positive and negative patterns. (F) SEM image obtained after electron beam patterning, along with EDS maps showing the elemental distribution in the patterned region. Reproduced with permission [209]. Copyright 2008, ACS. (G) Schematic illustration of soft lithography using a PDMS stamp: (1) pattern design, (2) stamp fabrication by casting PDMS on a master mold, (5) pattern transfer onto a substrate, and (6) stamp removal. (H) Soft lithography process has been used to generate various epoxy nanopillars with the same height, *h* = 9 µm and *d* = w =750 nm. Reproduced with permission [210]. Copyright 2006, ACS.

Vertically Aligned nanopillar electrode fabrication methods (Figure 8) have garnered considerable interest due to their critical role in advancing bioelectronics, energy storage, and sensing applications. These vertically aligned nanostructures offer high surface area, excellent charge transport properties, and enhanced cell‐electrode interactions. To achieve well‐defined control over their shape, spacing, and material characteristics, several fabrication strategies have been established. Each fabrication approach offers distinct benefits and limitations regarding accuracy, scalability, and compatibility with different materials, which makes them adaptable for a wide range of practical applications (Table 3).

**TABLE 3:** Comparison of representative fabrication methods for nanopillar structures and devices [165].

Technique	Definition	Resolution	Advantages	Disadvantages
Photolithography	Use light and a photomask to pattern a photoresist [181].	1 µm [182].	▪ Simple process flow [183]. ▪ Efficient [183]. ▪ High throughput through parallel processing [184].	▪ Requires cleanroom facilities [185]. ▪ Resolution limited by optical diffraction [186]. ▪ Requires flat substrates [185].
Electron beam lithography	Use electrons to pattern a resist [187].	>10 nm [188].	▪ Suitable for scalable fabrication [189]. ▪ Capable of producing complex geometries [189].	▪ Beam exposure may damage the substrate [190]. ▪ Proximity effects can generate unintended features [191]. ▪ High cost and process complexity [190]. ▪ Low throughput [190].
Focused ion beam lithography	Uses ions to pattern a resist [192].	>10 nm [193].	▪ Reduced proximity effects compared with EBL [160]. ▪ Lower forward scattering and radiation damage than EBL [187, 190].	▪ Ion beam exposure may damage the sample [194]. ▪ Sputtering can introduce surface defects [194]. ▪ Low throughput and long processing time [194].
Soft lithography	Uses an elastomeric mold (stamp) to fabricate and replicate structures [195].	35 nm [196]	▪ Compatible with fragile substrates [197]. ▪ Can incorporate biomaterials [197].	▪ Requires mold fabrication before replication [197]. ▪ Typically performed in a cleanroom environment [198]. ▪ Relatively high initial setup cost [198]. ▪ Demolding may damage the master structure [198].
Porous anodic aluminum templates (PAA)	Electrochemical process that forms self‐organized, highly ordered nanoporous anodic aluminum oxide [199].	10–100 nm (pore diameter) [199].	▪ Simple, rapid, and cost‐effective [200]. ▪ Enables wafer‐scale fabrication with high pore density [201].	▪ Often requires removal of the amorphous barrier layer [202]. ▪ Template removal may introduce structural defects [202].
Porous silicon templates (pSi)	Anisotropic etching of silicon at cryogenic temperatures to form high‐aspect‐ratio nanowire or trench arrays [203].	Down to 30 nm (diameter) [203].	▪ High aspect ratio structures (>100) [203]. ▪ Liquid‐free processing prevents stiction or collapse [203]. ▪ Highly CMOS compatible [203].	▪ Requires specialized cryogenic/vacuum equipment [203]. ▪ Results are influenced by feature‐size‐dependent loading effects [203].

### 5.1 Lithography Technique

Lithography is a fabrication method that allows for the creation of patterns on a substrate [204]. One of the most effective techniques to fabricate micro/nanopillar arrays is via the lithographic molding technique [205]. Photolithography (PL) constitutes one of the most prevalent lithographic techniques where light is applied onto a photosensitive (photoresist) through a mask to create patterns [206]. Another technique is electron beam lithography (EBL) which is a process in which electrons are used to scan a surface coated with a sensitive resist [207]. The electron beam is controlled to generate structures in certain regions.

The increasing demand for enhanced device performance and high‐throughput manufacturing has driven the development of diverse patterning methods at the sub‐micrometer scale. However, the practical implementation of many of these techniques remains challenging due to the absence of simple and scalable fabrication procedures suitable for large‐area production. To overcome these limitations, novel lithographic strategies have been introduced to enable cost‐effective, high‐fidelity patterning over a broad range of length scales. These methods are compatible with various functional materials and offer promising solutions to current challenges in microfabrication, thereby supporting continued technological advancement.

#### 5.1.1 Photolithography

Photolithography (PL) is a top‐down manufacturing method (Figure 8A), where a light‐sensitive material called a photoresist is subjected to light in specific areas to produce the desired structure. Photoresists are ultraviolet UV‐sensitive materials generated from polymers, sensitizers, and solvents [211]. Polymers undergo structural alterations when exposed to UV radiation. Moreover, sensitizers influence the solubility of the photoresist, while solvents alter the viscosity to facilitate straightforward application on the employed substrate. There are two primary types of photoresists: positive photoresists, which are exposed to UV radiation resulting in a soluble state, and negative photoresists, which turn into insoluble [181]. The photoresist is usually applied to the center of a chip covered with an oxide layer that serves as a barrier preventing impurity diffusion. The wafer is coated with a uniform, thin layer employing spin‐coating, where the layer's thickness is inversely related to the square root of the spinning speed [212]. Afterward, the wafer is subjected to a soft baking procedure to eliminate any remaining solvent, stabilize the photoresist, and enhance its adhesion. Once cooled, a pattern is created on the wafer utilizing a photomask, which blocks UV light except for specific clear spots that allow light to pass through. Photomasks are positioned with the wafer either via a mask aligner or fiducial markings, and the UV light interacts alongside the photoresist generating the intended design on the wafer [213]. Additionally, a mixed lithography approach combining electron beam lithography and photolithography enables the fabrication of vertically aligned nanopillar (Figure 8B‐D) or nanohole arrays alongside micrometer‐sized structures within a single layer [208]. Silicon nanopillars around 200 nm in diameter were obtained by this combination. Moreover, photoresist nanopillars have been fabricated via two‐photon lithography, with diameters ranging from 120 to 430 nm and heights varying from 330 to 1315 nm [214], whereas other scientists utilize nanosphere photolithography to create features smaller than 250 nm [215].

#### 5.1.2 Electron Beam Lithography

Electron beam lithography (EBL) is a maskless technology (Figure 8E), which exhibits similarities with photolithography [216]. EBL is a method that requires utilizing whether a positive or negative resist. However, instead of being affected by the presence of UV light, the resist's solubility is influenced by being subjected to a beam of electrons [207]. The wafer is scanned by the electron beam using two distinct methods: raster scanning and vector scanning. The wafer is partitioned into pixels, and the beam systematically moves across each horizontal line in a left‐to‐right direction during the raster scanning process. After analyzing the entire surface, the scanning process returns to the left‐hand side of the wafer and starts a new line. The scanning only activates when contact is necessary. Vector scanning involves dividing the image into vectors that represent the characteristics. Rather than scanning the whole wafer, the electron beam transfers from one feature to another [187]. The photomasks are composed of a layer of chrome that is applied onto a quartz substrate. The chrome layer is then carefully removed through etching to produce the desired pattern. This pattern will later be transmitted to the photoresist. The quartz substrate is utilized because it allows for the transmission of electrons [217].

Unlike the photolithography technique, EBL may achieve precise resolution as low as 10 nm, because electrons have a significantly shorter wavelength compared to photons [188]. A major limitation of EBL is the proximity effect. During electron beam exposure, incident electrons interact with the atoms and electrons of the resist, leading to beam deviation and the generation of forward‐scattered electrons. As a result, pattern resolution and dimensional accuracy can be adversely affected. In addition, EBL is a complex and costly technique that requires substantial maintenance and often suffers from low throughput, operating several orders of magnitude more slowly than conventional optical lithography methods [190].

The EBL method has been employed for the precise fabrication of vertically aligned nanopillar arrays. Diverse vertically aligned nanopillar types can be fabricated with EBL, including silicon [218] and gold [219] architectures. A two‐layer exposure technique has been utilized to promptly fabricate extensive silicon nanopillar arrays with diameters of 150 nm [218]. EBL has been integrated with reactive ion etching to fabricate silicon vertically aligned nanopillars with diameters under 100 nm and enhanced aspect ratios for the investigation of bacterial adhesion [220]. Furthermore, gold vertically aligned nanopillar arrays have been generated via EBL and electroplating, illustrating their applicability as biosensors as [219]. EBL is an efficient approach for constructing palladium nanowires with precise control over their size and structure. Using palladium hexadecylthiolate as a negative‐tone resist, nanowires as small as 30 nm in width have been successfully fabricated (Figure 8F) [209].

#### 5.1.3 Focused Ion Beam Lithography

Focused Ion Beam lithography (FIBL) is a top‐down fabrication method that utilizes an ion beam to etch the surface of a material, employing polymethylmethacrylate (PMMA) as a resistor [221]. The Gallium (Ga) ion source is utilized extensively because of its high atomic mass and low vapor pressure, melting temperature, and volatility [222]. At the top of the FIB column, there is a liquid reservoir containing Ga. This Ga serves to moisten a sharp Tungsten tip. An elevated voltage generates a strong electric field, resulting in the formation of a distinct cone‐like structure through the Ga material. Subsequently, the Ga atoms undergo ionization due to the electric field, and the ions are pushed down the column due to the elevated voltage [192]. The Ga ions interact with the surface of the intended material and remove the surface atoms, resulting in the milling of the surface and the formation of a structure.

Both EBL and FIBL offer a resolution below 10 nm [193]. However, FIBL offers certain advantages over EBL such as the proximity effect, which is significantly reduced due to the higher mass of ions compared to electrons, resulting in a decrease in backscattering. Nevertheless, FIBL has the potential to harm the material close to the ion beam, and metal atoms that have been ejected from the specimen can be redeposited in different regions, resulting in undesired defects or impurities. Contrary to EBL, the duration of the FIBL process is determined by the quantity of milling needed. Consequently, for large arrays, employing FIBL might not represent the most appropriate method of production [194]. 3D FIB lithography facilitates the production of vertically aligned nanopillar spintronic devices using metallic multilayer heterostructures [223]. Also, vertical Gaussian pillar nanostructures with high order on silicon were fabricated in a single step using FIB, demonstrating antireflective characteristics [224].

#### 5.1.4 Soft Lithography

Soft lithography employs an elastomeric stamp (Figure 8G), to produce and duplicate patterns. This is accomplished by methods including molding, printing, and embossing, achieving a resolution of around 35 nm [195]. Soft lithography has several advantages over traditional patterning techniques. These include a reduced cost, a less complicated setup, and increased throughput [196]. Additionally, soft lithography enables patterning with resolution spanning from the nanometer to micrometer scale. One limitation of this technique is that fabrication of the stamp master typically requires an additional lithographic method, such as photolithography or electron beam lithography. However, this step is generally performed only once, since the master can be reused multiple times to produce replica stamps [196]. The polymer poly(dimethylsiloxane) PDMS is frequently exploited for mold fabrication because of its flexible properties, transparent nature, hydrophobic characteristics, biocompatibility as well as capability to allow the transport of gases. Soft lithography techniques provide economical approaches for the fabrication of vertically aligned nanopillars utilizing diverse materials and dimensions. Silicon nanopillars with aspect ratios of 2.7 and diameters of around 200 nm can be fabricated by polymethylmethacrylate (PMMA) patterning together with reactive ion etching (RIE) [225]. Polymeric nanopillars, with diameters between 300 nm and 1 µm, can be fabricated using replica molding, where harder materials acquire greater aspect ratios as shown in Figure 8H [210].

### 5.2 Template‐Assisted Synthesis

**FIGURE 9:**
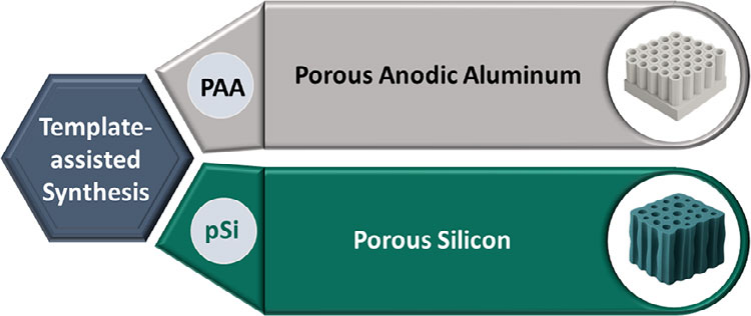
A schematic to illustrate the main common types of template‐assisted synthesis including porous anodic alumina PPA and porous silicon pSi.

Template‐assisted synthesis is a fundamental strategy for the controlled fabrication of nanostructured materials. The preparation of both simple and complex low‐dimensional nanostructures largely relies on templates with predefined dimensions, architectures, and physicochemical properties. This approach enables precise control over the size, morphology, arrangement, and growth direction of nanostructured materials, which is often difficult to achieve through template‐free methods [226]. In principle, a wide range of current synthetic methodologies, either individually or in combination, can be applied in template‐assisted nanomaterial synthesis. The general procedure typically involves three main steps: preparation of the template, synthesis of the target material using the template as a scaffold, and, when necessary, removal of the template through physical or chemical means. Templates may include a variety of nanostructured materials, such as nanowires, nanopillars, nanotubes, block copolymers, and porous matrices. In this section, the use of porous anodic alumina (PAA) and porous silicon (pSi) templates for the fabrication of nanowire/nanopillar electrodes is discussed (Figure 9).

#### 5.2.1 Porous Anodic Aluminum (PAA)

**FIGURE 10:**
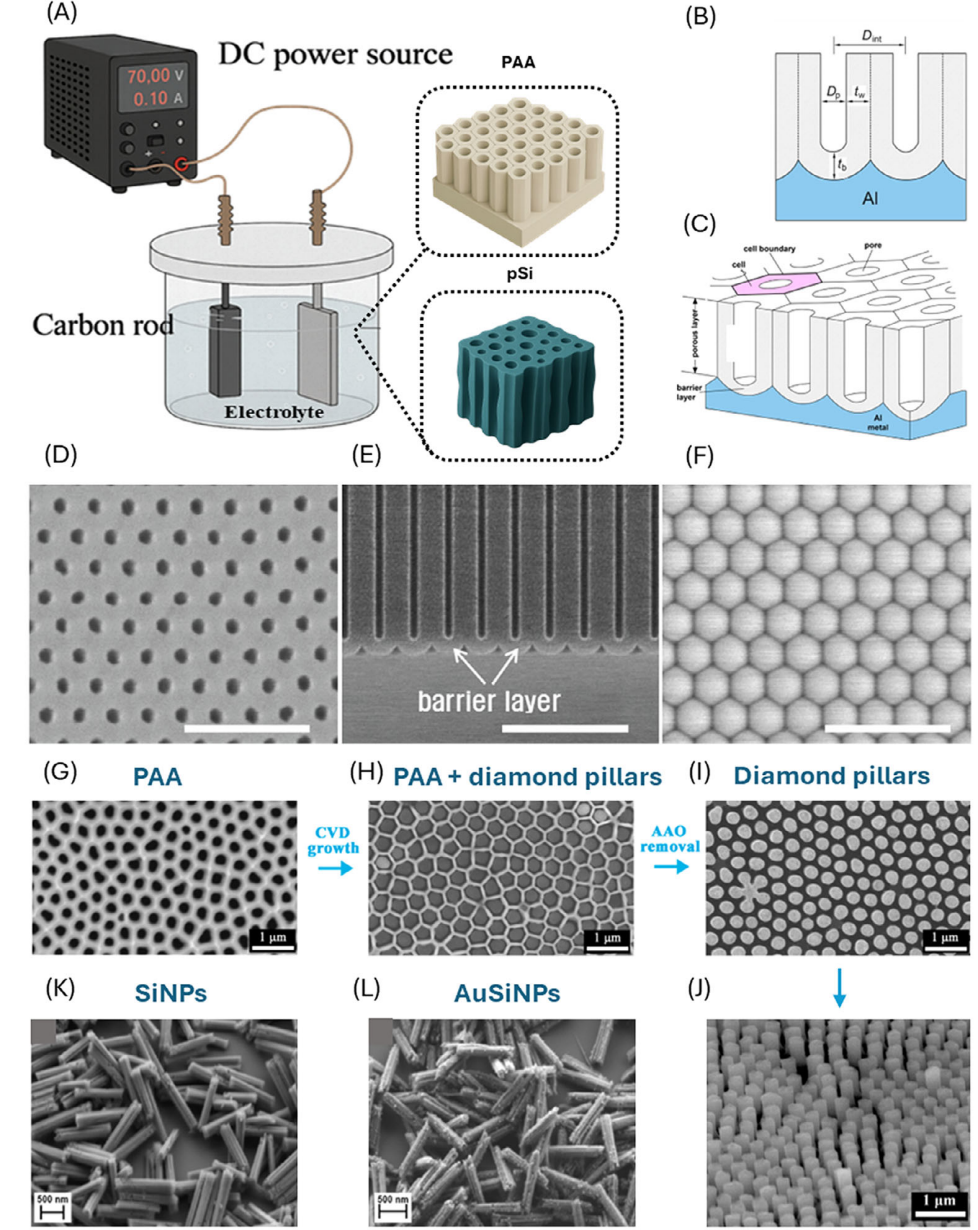
Schematic structure of (A) Anodization setup for template‐assisted synthesis. (B) A cross‐sectional view of PAA template. Reproduced with permission [226]. Copyright 2014, ACS. (C) A honeycomb‐like structure of PAA. Reproduced with permission [226]. Copyright 2014, ACS. (D–F) SEM images illustrate various perspectives of PAA: (D) the top surface, (E) the barrier layer, and (F) the bottom surface. Scale bars in all SEM images represent 1 µm. Reproduced with permission [226]. Copyright 2014, ACS. Top‐view SEM images of (G) PAA template, (H) diamond pillars within PAA template, and (I) released diamond pillars. (J) SEM image from tilted side of the obtained diamond pillars. Reproduced with permission [234]. Copyright 2023, MDPI. SEM images of (K) SiNPs deposited on Si surface, (L) AuSiNPs deposited on Si surface. Reproduced with permission [235]. Copyright 2017, ACS.

The use of nanoscale templates in template‐assisted synthesis enables precise control over key properties of nanostructured materials, including their size, morphology, and growth direction. As a result, diverse nanostructures (Figure 10) have been effectively synthesized, including nanotubes [227], nanowires [228], as well as arranged arrays of vertically aligned nanopillars [229] In comparison to alternative approaches for synthesizing 1D nanostructures, the PAA templating process offers significant advantages. The 1D nanostructures produced by this templating method include a wide array of materials, including carbon [230], metals [231], oxides [232], semiconductors [233], and polymers [231]. Self‐ordered porous anodic alumina PAA oxides can be generated by precisely controlling the anodization process of aluminum substrates. The PAA templates are generated by electrochemically anodizing pure Al that has been previously electropolished as shown in Figure 10A.

The two‐step anodization process, as described by Masuda et al. [236], involves anodizing aluminum to form an oxide layer, removing distortions, and then re‐anodizing the concave substrate to create ordered nanoporous structures. Parameters such as voltage, electrolyte composition, and temperature allow precise control of vertically aligned nanopillar size and density. PAA films grown on aluminum consist of a thin barrier oxide layer and thick porous oxide film with parallel nanopores as shown in Figure 10B [226]. The structure of porous PAA is like a honeycomb. On an Al base with a porous oxide layer, there are a lot of reciprocally parallel pores. Each round nanopore and its ambient oxide make up a hexagonal cell (Figure 10C) that is lined up with the surface of the metal. A nearly hemispherical morphology (Figure 10D) with a thin barrier layer (Figure 10E) of oxide is attached to each nanopore at the interface of metal/oxide for closing purposes (Figure 10F). Cell organization is performed automatically under appropriate anodizing conditions to create a hexagonal structure. The amount of charge involved in electrolytic oxidation determines how thick the porous PAA layer is [237]. Consequently, adjusting the depth of the nanopores depends on the time of anodization [238].

##### 5.2.1.1 Electrochemical Deposition (ECD)

Electrochemical deposition (ECD) of metal within a track‐etched mica template technique has been initially developed to produce Sn, In, and Zn nanowires [239]. The ECD of materials into the pores of a PAA template offers significant benefits compared to alternative approaches for generating nanowires or nanotubes. In comparison to other deposition methods, including CVD, PVD, and atomic layer deposition (ALD), ECD onto porous templates is straightforward, cost‐effective, and can be performed without sophisticated equipment. In addition, nanowires of various materials, including gold, nickel, and silver, can be synthesized within PAA templates using electrochemical deposition or electroless deposition techniques [240, 241, 242]. Silver telluride and silver nanowires have been effectively synthesized using electrodeposition using porous anodic alumina templates. Ag2Te nanowires with adjustable composition were synthesized from DMSO solutions, displaying a monoclinic structure and smooth surfaces [243].

##### 5.2.1.2 Physical Vapor Deposition (PVD)

Physical vapor deposition (PVD) is a technique employed for depositing thin films of materials onto substrates. The process involves transforming a solid material into a vapor state and then depositing it onto a substrate to create a thin film through condensation [112]. Physical vapor deposition has two primary types of deposition process which are thermal or vacuum evaporation and sputtering evaporation [244]. Various hybrid procedures and variations have been obtained within these two primary types of PVD. PVD has traditionally been employed for depositing electrically conductive films [245] and corrosion‐resistant coatings [246]. PVD is widely employed due to its versatility in accommodating a broad range of coating materials, as well as its ability to deposit thin films on diverse substrates. The development of ordered Ni nanostructures on glass substrates involves the anodization of aluminum films to produce porous alumina templates, succeeded by direct current electrodeposition of Ni into the nanopores [247] This method generates Ni nanowires with diameters between 18 and 180 nm and lengths from 0.5 to 2.6 µm, demonstrating considerable magnetic anisotropy relative to their aspect ratios. ZnO nanowires have been successfully synthesized using physical vapor deposition techniques, demonstrating promising optical properties [248]. Oblique angle PVD can produce aluminum‐niobium Al–Nb alloy nanopillars with shapes ranging from nanohorns to nanoplates, depending on composition and deposition angle [249].

##### 5.2.1.3 Chemical Vapor Deposition (CVD)

Chemical vapor deposition (CVD) involves the formation of a solid material on or near the substrate surface through chemical reactions that lead to thin‐film growth. The mechanisms governing CVD differ substantially from those of PVD. In PVD, thin films are formed through the condensation of atoms or molecules onto the substrate surface following evaporation or sputtering. By contrast, CVD is a more complex process that relies on chemical reactions under carefully controlled conditions, including temperature, pressure, reaction kinetics, and the transport of momentum, mass, and energy [112]. The efficacy of the films generated is influenced by the interactions of several transport mechanisms and chemical reactions that occur in the CVD chamber. These interactions are dependent on process parameters such as flow rates, pressure, temperature, concentration of chemical species, and reactor geometry [250]. CVD is distinguished by its ability to produce both simple and complex compounds with simplicity, typically at low temperatures. CVD methods can produce a wide range of 1D nanomaterials, including carbon nanotubes and semiconductor nanowires, with potential applications in composite materials and heterostructure devices [251]. Pulsed electrodeposition throughout porous alumina templates produces densely packed monocrystalline silver nanowire with diameters ranging from 30 to 70 nm [252]. CVD has been used to grow diamond nanopillar arrays on ultrathin AAO membranes, resulting in ordered structures with diameters of about 325 and 85 nm as shown in Figure 10G–J [234].

##### 5.2.1.4 Spin Coating

Spin coating is a widely used technique for depositing uniform thin films onto flat substrates. In this process, a small volume of coating solution is placed at the center of the substrate, which may initially be stationary or rotating at a low speed. The substrate is then spun at high speed, allowing the coating material to spread across the surface under centrifugal force. Rotation is maintained until the desired film thickness is achieved. Because the solvent is typically volatile, it evaporates rapidly during spinning. As a result, higher angular velocities generally produce thinner films. The film's thickness is reliant upon the viscosity and concentration of both the solution and the solvent. Spin coating is extensively employed in microfabrication for producing thin films with thicknesses under 10 nm. It is employed extensively in photolithography to deposit layers of photoresist around 1 µm in thickness [253].

Microporous silica was deposited on an anodic alumina template by applying a mesoporous silica layer with a pore size of 2–6 nm through dip coating on the anodized disc. Subsequently, the mesoporous gas separation membrane layer is applied via spin coating, resulting in defect‐free mesoporous silica with enhanced selectivity and permittivity [254] The synthesis of single crystalline PbO2 nanowires was conducted by a spin‐coating procedure involving the application of a lead oxide sol‐gel solution onto a PAAM substrate [255].

#### 5.2.2 Porous Silicon (pSi)

Porous silicon (pSi) has been widely explored as a high‐performance platform owing to its excellent biocompatibility, surface tunability, and reproducibility [256]. Its unique optical, electrical, and electrochemical properties offer broad opportunities for the development of advanced devices and enable efficient transduction of electrical signals. Furthermore, its considerable surface area offers an outstanding foundation for the identification of biomarkers. Anodic electrochemical etching of a single crystalline silicon chip in an aquatic fluoride‐based electrolyte, particularly hydrofluoric acid is an increasingly typical method for manufacturing pSi. In most cases, a platinum electrode serves as the cathode and a wafer of silicon serves as the anode as shown in Figure 10A [256, 257]. Silicon wafers develop pores as a result of the selective breakdown of their crystalline structures caused by a current bias traveling between the anode and cathode. Manufacturing parameters including current density, silicon wafer type, doping level, and electrolyte nature influence the engineering of the pSi structure. The structure of pSi is characterized by a sponge‐like network of nanoscale pores formed on a silicon substrate.

A wide range of materials including metals, semiconductors, oxides, carbon‐based compounds, and conductive polymers can be deposited into the cylindrical pores of pSi templates using techniques such as sol‐gel processing, vapor deposition, and electrochemical deposition. These methods enable the formation of diverse nanostructures, including nanowires, nanotubes, nanodots, nanorods, and other heterogeneous morphologies. Pore morphology, including porosity and pore size, can be adjusted to suit different applications by modifying these parameters. The porous structure allows for atomic layer deposition of materials like ZnO and TiO, creating nanocomposite pillars with potential applications in photocatalysis and sensing [258]. The visible photoluminescence observed in these structures is attributed to Si nanocrystals decorating the pillar surfaces, with emission characteristics dependent on oxidation and temperature [259]. A study has investigated gold‐coated pSi nanostructures for the targeted hyperthermal therapy of bacterial infections [235]. Alhmoud et al. developed gold nanoparticle‐decorated porous silicon nanopillars (Figure 10K,L) that demonstrated excellent photothermal conversion properties and reduced bacterial viability by up to 99% when irradiated with near‐infrared (NIR) light. Porous silicon and its nanoparticles have gained attention in biomedical applications due to their biocompatibility, biodegradability, and high surface area, making them promising platforms for drug delivery, diagnostics, and therapy [260]. These properties collectively position porous silicon vertically aligned nanopillars as valuable materials for various applications.

## 6 Use of Vertically Aligned Nanopillar Electrodes for Electrophysiology and Cell‐Electrode Coupling

Vertically aligned nanopillar architectures have emerged as promising substrates for electrophysiological applications. Their incorporation as electrode structures can substantially increase the effective surface area, improve adhesion, and enhance the stability of bioelectronic interfaces, thereby making them highly attractive for long‐term neural and optical applications. Complementing this approach, one study systematically evaluated the stability, adhesion, and biocompatibility of planar electrodes composed of indium tin oxide (ITO), Au, Ag, and Al in various aqueous and biological media [261]. The results reported that ITO maintained excellent conductivity and transparency in all conditions, while Au and Ag on glass exhibited rapid delamination, which was significantly reduced by adding an SU‐8 adhesion layers. In contrast, Al was prone to oxidation and surface roughening, and long‐term exposure caused wrinkling of the SU‐8 layer. Biocompatibility tests using Human Embryo Kidney (HEK293) cells confirmed no cytotoxic effects for ITO, Au, or Al, whereas polyethylenimine PEI showed strong toxicity.

**FIGURE 11:**
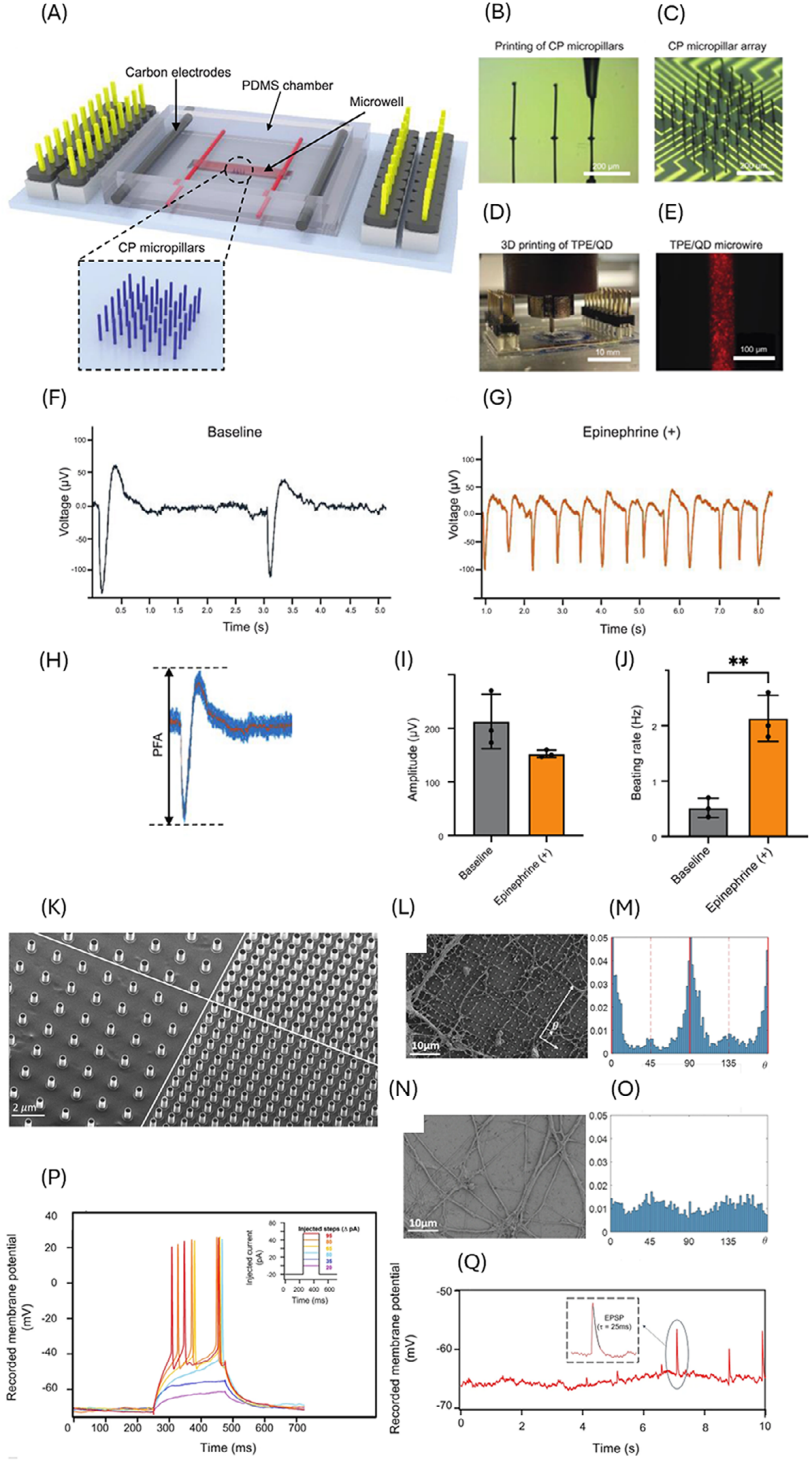
(A‐E) Illustration and characterization of a multifunctional heart‐on‐a‐chip device. Reproduced with permission [262]. Copyright 2023, IOP Publishing. (A) Schematic showing the system components. (B) Direct writing of a single CP micropillar (optical image; scale bar, 100 µm). (C) Microscopy view of the micropillar array (scale bar, 200 µm). (D) 3D‐printed TPE/QD nanocomposites positioned beside the microwell (optical image; scale bar, 10 mm). (E) Fluorescent image highlighting the nanocomposite microwire (scale bar, 100 µm). (F–J) Extracellular potential recordings of cardiac microtissues on the microelectrode‐based device. Reproduced with permission [262]. Copyright 2023, IOP Publishing. Representative traces under spontaneous beating (F) before and (J) after epinephrine stimulation. (H) Schematic of a typical peak and (I) corresponding field potential amplitude (FPA). (J) Beating rate quantification before and after epinephrine treatment (t‐test, *n* = 3, mean ± s.d. **p < 0.01). (K) SEM image of diamond pillar arrays positioned in adjacent square patterns. Reproduced with permission [263]. Copyright 2023, Springer Nature. (L‐O) Preferential neurite orientation of primary hippocampal neurons on nanopillar arrays. Reproduced with permission [263]. Copyright 2023, Springer Nature. (L and M): nanostructured substrate, (N and O): flat surface. (L and N) SEM images at 4.5k×. (M and O) Histograms of axon direction relative to a principal grid axis. Red lines indicate the two orthogonal nearest‐neighbor directions. Each histogram is based on analysis of 15 images. (P) Action potentials recorded from neurons on the diamond nanostructure using incremental depolarizing pulses, exhibiting overshoot and hyperpolarization. Reproduced with permission [263]. Copyright 2023, Springer Nature. (Q) Spontaneous Excitatory post synaptic potentials (EPSPs) recorded at −65 mV; inset fit yields τ = 25 ms. Reproduced with permission [263]. Copyright 2023, Springer Nature.

Nano pillar arrays have been engineered for several applications due to their distinctive characteristics arising from their significant feature size and structural periodicity (Figure 11). Recent improvements have resulted in a versatile nanoelectrode device that can record intracellular action potentials from many cell types, including cardiomyocytes, neurons, and bacterial biofilms, with excellent resolution over long durations [264]. Vertically aligned nanopillar electrodes are designed to provide simultaneous, long‐term, multisite electrophysiological intracellular recording and stimulation of neurons to enhance the understanding of the correlations between neural circuit connectivity and function [265]. Recent developments in heart‐on‐chip technology have facilitated the continuous evaluation of cardiac tissue performance. These platforms incorporate conductive hydrogel pillars [266] or 3D‐printed microelectrodes [262] (Figure 11A–J) for real‐time electrophysiological recordings and contractile force assessments. In addition, diamond nanopillar arrays (Figure 11K–Q) offer a biocompatible platform with highly improved sensing potential for neural research [263]. This study demonstrated that primary mouse hippocampal neurons can successfully grow on high‐aspect‐ratio nanopillars, aligning along the grid axes and forming intimate membrane‐pillar interfaces. Such interactions enhance sensitivity in quantum sensing platforms based on nitrogen‐vacancy (NV) centers, enabling improved monitoring of mammalian neuronal activity. Vertical nanopillar electrode arrays are effective platform for intracellular interfacing with neuronal circuits, providing scalability and enabling high‐resolution recordings [87]. In this study, vertical NWs developed featuring 150 nm in diameter and 3 µm in height, having a degenerately doped Si core encased in a SiO_2_ shell, with the ends coated with Ti/Au. A standard network of dissociated rat cortical neurons cultured on a VNEA after five days in vitro. Vertical nanopillar electrodes enable long‐term recording of both extracellular and intracellular action potentials in cardiomyocytes, with the ability to switch between recording modes through nanoscale membrane permeabilization. The effectiveness of vertical nanowire microelectrode arrays (MEAs) was successfully demonstrated through multi‐site recording and stimulation of neural networks [20]. In this study FIB deposition was used to place vertical Pt NWs, with diameters of 150 nm and heights of 1–2 µm, onto microelectrodes. These findings indicate that vertical NWs can effectively bridge the interface between microelectrodes and cells, providing direct access to intracellular data.

In addition, considerable developments in electrode array technologies have emphasized the fabrication of soft and flexible interfaces that closely align with the mechanical characteristics of biological tissues [267]. In this study, a conductive hydrogel micropillar electrode array with a tissue‐like Young's modulus was developed, resulting in a significant enhancement in both signal amplitude and SNR compared with conventional electrode rigids. Likewise, another research group engineered a flexible micropillar electrode array with nanoscale surface roughness, which facilitated effective electrical interfacing with neural tissues [268]. Recent research has investigated the application of silicon micro‐pillar substrates (MPS) for the culturing of dorsal root ganglion (DRG) neurons. These 3D platforms enhance neuronal development and neurite alignment comparable to conventional glass surfaces. The electrophysiological characteristics of DRG neurons on MPS were comparable to those on planar surfaces, exhibiting no significant variations in action potential parameters or firing patterns [269].

Vertically aligned nanopillar electrodes were developed as a practical device for intracellular electrophysiological studies across diverse cell types. These vertical nanostructures provide high‐quality, long‐term recordings of action potentials from individual cells and cell networks [270]. Vertically aligned nanopillar arrays can be manufactured through various approaches such as template‐assisted procedures and microfabrication processes, offering scalable and adjustable manufacturing [270, 271]. Vertically aligned nanopillar electrodes provide enhanced cell‐electrode coupling, reduced impedance, and superior signal quality compared to planar electrodes [271, 272]. Moreover, vertically aligned nanopillar‐based dry electrodes demonstrate potential for wearable ECG monitoring, presenting reduced contact impedance and enhanced SNR relative to planar electrodes [272]. Furthermore, with an established understanding that nuclear mechanics and mechanotransduction regulate core cellular functions (e.g., migration, proliferation, polarization) and that pathological alterations in nuclear mechanics contribute to disease [273, 274, 275, 276, 277]. Vertically aligned nanopillar arrays provide a uniquely controllable interface to probe these properties in situ within adherent, functioning cells. In contrast to bulk mechanical assays (e.g., AFM, micropipette aspiration) that typically report whole‐nucleus or whole‐cell responses, nanopillars impose highly localized, subcellular deformations that are generated and sustained within intact cells, enabling access to deformation modes that are closer to those encountered in vivo at submicron length scales. Hanson et al. [278] demonstrate that membrane deformation around nanopillars can be mechanically relayed through the cytoskeleton to produce nanopillar‐indexed nuclear envelope indentations, and that the magnitude/shape of these deformations reports on nuclear stiffness and cytoskeletal balance (e.g., actin‐mediated pulling versus intermediate filament resistance). Importantly, nanopillar geometry provides an experimentally tuneable “mechanical boundary condition”: deformation depth, width, and curvature can be systematically controlled by pillar radius, pitch, and height, supporting quantitative mechanical inference rather than purely phenomenological observations.

Beyond acting as mechanical perturbators, vertically aligned nanopillars also function as integrated sensors that can combine localized optical readouts with electrical interfacing. Xie et al. [279] show that transparent SiO_2_ nanopillars embedded in a non‐transparent metal layer generate evanescent excitation along the pillar surface, yielding a highly confined illumination volume that extends into the cell interior (on the order of hundreds of nanometers) and enabling localized fluorescence measurements inside live cells while maintaining long‐term culture viability and tight membrane wrapping. In parallel, the intimate membrane‐nanopillar interface that supports optical confinement is also highly advantageous for electrical coupling, for example, by improving seal resistance and reducing the cell‐electrode separation distance. This makes such platforms particularly suitable for correlative electromechanical investigations, in which optical assessment of membrane or nuclear deformation and cytoskeletal organization can be directly integrated with electrical measurements probing the membrane state at the nano‐bio interface. Collectively, these studies highlight vertically aligned nanopillar arrays as a scalable platform for investigating cellular mechanics through combined optical and electrical modalities, where geometry‐controlled intracellular perturbations can be monitored with high spatial precision and correlated with mechanotransduction processes in living cells.

## 7 Conclusion and Future Perspectives

Vertically aligned nanopillar electrodes represent a significant advancement with substantial potential for a wide range of applications, particularly in the field of electrophysiological sensing. These electrodes demonstrate exceptional properties, such as high conductivity, superior biocompatibility, and the ability for customization for specific purposes, making them essential instruments in both invasive and non‐invasive applications. The characteristics of these nanostructures, including adhesion, breathability, biodegradability, and flexibility, enhance their adaptability in medical devices and biosensing technologies.

The well‐established fabrication technologies, such as lithography and template‐assisted procedures, offer precise control over the electrode structure, thereby ensuring their efficiency and performance along with mass‐fabrication. Moreover, innovations in materials including metal‐based, carbon‐based, and polymer‐based vertically aligned nanopillars present enhanced opportunities for customization and functionality. As fabrication techniques and materials advance, the incorporation of vertically aligned nanopillar electrodes into practical applications will further expand the scope of bioelectronics, facilitating future advancements in various applications.

In addition, emerging the AI‐assisted nanofabrication and machine learning‐driven optimization reshaping the design of vertically aligned nanopillar electrodes for advanced sensing applications. Al‐driven algorithms now support real‐time signal analysis and predictive diagnostics. By integrating the machine learning with nanofabrication techniques, researchers can automate material selection, enhance precision, and minimize the structural defects. This integration not only improves material reliability and efficiency but also supports the development of energy‐efficient and intelligent electronics for wearable and implantable devices. AI‐driven optimization can fine‐tune the structure, composition, and geometry of vertically aligned nanopillar electrodes, improving their stability, sensitivity, and biocompatibility.

The growing use of wearable and clinical electrodes leads to the disposal of millions of single‐use polymer‐based units annually, most of which accumulate in landfills due to the difficulties associated with their composition, which cannot be easily recycled. The persistence of plastic substrates, the release of silver residues, and the loss of valuable metals highlights the needs for environmentally friendly electrode technologies. Vertically aligned nanopillar electrodes emerge as strong candidates because the offer high signal performance while requiring for less material and can be manufactured from biodegradable metals or transient silicon structures. Although progress in sustainable bioelectronics is ongoing, there remains significant potential to design nanostructured electrodes particularly for biodegradation and recycling.

In addition to reducing electronic waste from disposable polymer‐based interfaces, future bioelectronic platforms may increasingly benefit from transient and bioresorbable materials systems. Although reports on fully transient nanopillar electrodes are still limited, the broader state of the art already includes bioresorbable silicon‐based electronics for transient cortical electrophysiological mapping and fully bioresorbable hybrid opto‐electronic neural implants for simultaneous recording and stimulation, demonstrating that high‐performance neural interfaces can be designed to safely disappear after use [280, 281]. Related progress in magnesium‐based bioresorbable electrodes and stimulators further highlights the potential of degradable metals for temporary implantable bioelectronics [282, 283]. These advances suggest a promising pathway toward more sustainable, clinically practical, and environmentally responsible next‐generation nanostructured electrode technologies.

## Funding

This work was supported by the NHMRC Early Career Fellowship (GNT1143296), Australian Research Council (DP200102723), and the UNSW Scientia Grant, with additional support from the Australian Centre for NanoMedicine, UNSW. Funding for Mohammad Alzahrani was provided by the Saudi Arabian Cultural Mission (SACM) and King Khalid University (KKU), KSA.

## Conflicts of Interest

The authors declare no conflicts of interest.

## Data Availability

The data that support the findings of this study are available from the corresponding author upon reasonable request.
